# *In silico* Comparison of 19 *Porphyromonas gingivalis* Strains in Genomics, Phylogenetics, Phylogenomics and Functional Genomics

**DOI:** 10.3389/fcimb.2017.00028

**Published:** 2017-02-14

**Authors:** Tsute Chen, Huma Siddiqui, Ingar Olsen

**Affiliations:** ^1^Department of Microbiology, The Forsyth InstituteCambridge, MA, USA; ^2^Department of Oral Biology, University of OsloOslo, Norway

**Keywords:** comparative genomics, phylogenetics, phylogenomics, Porphyromonas gingivalis

## Abstract

Currently, genome sequences of a total of 19 *Porphyromonas gingivalis* strains are available, including eight completed genomes (strains W83, ATCC 33277, TDC60, HG66, A7436, AJW4, 381, and A7A1-28) and 11 high-coverage draft sequences (JCVI SC001, F0185, F0566, F0568, F0569, F0570, SJD2, W4087, W50, Ando, and MP4-504) that are assembled into fewer than 300 contigs. The objective was to compare these genomes at both nucleotide and protein sequence levels in order to understand their phylogenetic and functional relatedness. Four copies of *16S rRNA* gene sequences were identified in each of the eight complete genomes and one in the other 11 unfinished genomes. These 43 *16S rRNA* sequences represent only 24 unique sequences and the derived phylogenetic tree suggests a possible evolutionary history for these strains. Phylogenomic comparison based on shared proteins and whole genome nucleotide sequences consistently showed two groups with closely related members: one consisted of ATCC 33277, 381, and HG66, another of W83, W50, and A7436. At least 1,037 core/shared proteins were identified in the 19 *P. gingivalis* genomes based on the most stringent detecting parameters. Comparative functional genomics based on genome-wide comparisons between NCBI and RAST annotations, as well as additional approaches, revealed functions that are unique or missing in individual *P. gingivalis* strains, or species-specific in all *P. gingivalis* strains, when compared to a neighboring species *P. asaccharolytica*. All the comparative results of this study are available online for download at ftp://www.homd.org/publication_data/20160425/.

## Introduction

The Gram-negative anaerobic rod-shaped bacterium *Porphyromonas gingivalis* is one of the most important pathogens in chronic adult periodontitis (Socransky et al., 1998; Darveau et al., 2012; Hajishengallis et al., 2012). Colonization with *P. gingivalis* is also associated with some systemic diseases, including cardiovascular diseases, rheumatoid arthritis, and Alzheimer's disease (Demmer and Desvarieux, 2006; Lundberg et al., 2010; Olsen and Singhrao, 2015). It has become increasingly clear that strains of *P. gingivalis* differ in their pathogenicity and their ability to invade tissues and cells varies as much as three orders of magnitude (Dorn et al., 2000; Lundberg et al., 2010; Dolgilevich et al., 2011; Olsen and Progulske-Fox, 2015). Thus, W83 is considered a virulent strain while ATCC 33277 is less virulent. The AJW4 strain had the lowest invasion ability of 27 strains tested (Dolgilevich et al., 2011).

A comparative genomics study focusing on differences that affect virulence in a mouse model identified over 150 divergent genes (Chen et al., 2004). Dolgilevich et al. (2011) suggested deficiency in multiple genes as a basis for the *P. gingivalis* non-invasive phenotype. Actually, more than 100 genes were missing from the genome of a non-invading strain. The interstrain genomic polymorphisms and the individual host response have been suggested to be the key to disease initiation and progression (Dolgilevich et al., 2011). Genomic arrangement may also play a key role in the difference in virulence. For example, Naito et al. (2008) found that although the genome size and GC content were almost the same in strain ATCC 33277 and W83 there were extensive rearrangements between the two strains. *P. gingivalis* has been suggested to harbor many genetic mobile elements such as insertion sequence (IS), miniature inverted-repeat transposable element (MITE) and conjugative transposons CTns (Duncan, 2003; Naito et al., 2008; Tribble et al., 2013; Klein et al., 2015).

Together they are responsible for the fluidic genomic structure of this species (Naito et al., 2008; Tribble et al., 2013). The structural changes of the *P. gingivalis* genomes caused by these elements might have generated many strain-specific protein-coding sequences (CDs) and may have resulted in differences in various phenotypes including important virulence factors (Naito et al., 2008).

To date, a total of 19 *P. gingivalis* genome sequences have been published including eight completed (strains W83, ATCC 33277, TDC60, HG66, A7436, AJW4, 381, and A7A1-28); and 11 high-coverage draft sequences (JCVI SC001, F0185, F0566, F0568, F0569, F0570, SJD2, W4087, W50, Ando, and MP4-504) that are assembled into fewer than 300 contigs. These strains were isolated from various sources including the well-studied laboratory cultures with different degree of virulence, clinical samples from patients with different disease states, as well as an environmental strain isolated from a hospital bathroom sink drain. Together these sequences provide a great opportunity for a comparative genomics study and the results will provide valuable information to better understand the disease mechanism of this important periodontal pathogen. The aim of this study was to conduct *in-silico* genomics comparison for theses genomes using various approaches in the areas of phylogenetics, phylogenomics, and functional genomics. Results that we found most important and interesting are presented in this paper whereas complete results derived from this study are also made available for download online for further investigation.

## Materials and methods

### Sequence sources

Genomic sequences used in this study were downloaded from the NCBI FTP site (ftp://ftp.ncbi.nlm.nih.gov/genomes/all). The versions that were downloaded are also available online at ftp://www.homd.org/publication_data/20160425. A summary of all the meta information for each genome is available in the Excel file PG_Genome_Summary.xlsx in the above FTP folder. This file lists all the detail information that are provided by NCBI, such as methods for sequencing, assembling and annotation, as well as various IDs for the same genome including GenBank Accession, GenBank Assembly Accession, Refseq Accession, Refseq Assembly Accession. Table 1 lists the basic information and sources of the sequence data of the 19 *P. gingivalis* genomes analyzed in this report.

**Table 1:** **Summary of all the *P. gingivalis* genome sequences compared in this report^a^**.

**Strain**	**Sequence release date^b^**	**Genome size (bps)**	**Contigs**	**GenBank accession**	**Bioproject**	**Biosample^c^**	**Submitter**
W83	2003-09-02	2,343,476	1	AE015924	PRJNA48	SAMN02603720	*Porphyromonas gingivalis* Genome Project
ATCC_33277	2008-05-20	2,354,886	1	AP009380	PRJDA19051		Kitasato Univ.
TDC60	2011-05-23	2,339,898	1	AP012203	PRJDA66755		Tokyo Medical and Dental Univ.
W50	2012-06-25	2,242,062	104	AJZS01000000	PRJNA78905	SAMN00792205	J. Craig Venter Institute
JCVI_SC001	2013-04-24	2,426,396	1,284	CM001843^d^ APMB01000000	PRJNA167667	SAMN02436407	J. Craig Venter Institute
F0568	2013-09-16	2,334,744	154	AWUU01000000	PRJNA173937	SAMN02436723	Washington Univ.
F0569	2013-09-16	2,249,227	111	AWUV01000000	PRJNA173938	SAMN02436724	Washington Univ.
F0570	2013-09-16	2,282,791	117	AWUW01000000	PRJNA173939	SAMN02436747	Washington Univ.
F0185	2013-09-16	2,246,368	113	AWVC01000000	PRJNA198891	SAMN02436815	Washington Univ.
F0566	2013-09-16	2,306,092	192	AWVD01000000	PRJNA198892	SAMN02436881	Washington Univ.
W4087	2013-09-16	2,216,597	114	AWVE01000000	PRJNA198893	SAMN02436749	Washington Univ.
SJD2	2013-12-04	2,329,548	117	ASYL01000000	PRJNA205615	SAMN02470968	Shanghai Jiao Tong Univ. School of Medicine
HG66	2014-08-14	2,441,780	1	CP007756	PRJNA245225	SAMN02732406	Univ. of Louisville
A7436	2015-08-11	2,367,029	1	CP011995	PRJNA276132	SAMN03366764	Univ. of Florida
AJW4	2015-08-26	2,372,492	1	CP011996	PRJNA276132	SAMN03372093	Univ. of Florida
Ando	2015-09-17	2,229,994	112	BCBV01000000	PRJDB4201	SAMD00040429	Lab. of Plant Genomics and Genetics, Dept. of Plant Genome Research, Kazusa DNA Research Institute
381	2015-10-14	2,378,872	1	CP012889	PRJNA276132	SAMN03656156	Univ. of Florida
A7A1-28	2015-11-17	2,249,024	1	CP013131	PRJNA276132	SAMN03653671	Univ. of Florida
MP4-504	2016-02-09	2,373,453	92	LOEL01000000	PRJNA305025	SAMN04309157	Univ. of Washington

a *For a more detailed list of this table please follow this web link: ftp://www.homd.org/publication_data/20160425/*.

b *Genomes of this table are sorted by the original sequence release date*.

c *Unassembled raw sequence reads from which the assembly that was done can be traced back by the Biosample ID, if available*.

d *This Genbank number shows the sequence as “circular,” however it is a single pseudo-contig with many Ns filling the gaps. Thus, it should not be considered as a complete genome*.

### Strain information

#### W83, ATCC 33277, and W50

These most-studied laboratory cultures were among the first *P. gingivalis* strains sequenced. Strain W83 was isolated in the 1950s by H. Werner (Bonn, Germany) from an undocumented human oral infection and was brought to The Pasteur Institute by Madeleine Sebald during the 1960s. It was subsequently obtained by Christian Mouton (Quebec, Canada) during the late 1970s. W83 was reported to be also known as strain HG66 (Nelson et al., 2003), however it has been demonstrated that the two are very different strains based on data shown in this report. Strain W50 was originally isolated from a clinical specimen by H. Werner and first studied for known virulence (Marsh et al., 1994). W50 is also known as ATCC 53978 based on the description of the BioSample ID SAMN00792205 (http://www.ncbi.nlm.nih.gov/biosample/?term=SAMN00792205). The strain ATCC 33277 used for genomic sequencing was directly obtained from the American Type Culture Collection (ATCC) and was described as “has been kept for more than 20 years” by the authors (Naito et al., 2008).

#### TDC60

This strain was isolated from a severe periodontal lesion at Tokyo Dental College in Japan. Strain TDC60 exhibited higher pathogenicity in causing abscesses in mice than strains W83 and ATCC 33277 and other strains tested in the college (Watanabe et al., 2011).

#### JCVI SC001

This strain was not isolated from the human oral cavity; instead the genomic sequence was derived from single cells found in the biofilm of a hospital bathroom sink drain. The sequence was the first report of a human pathogen sequenced based on a single-cell genomic sequencing approach by capturing DNA from a complex environmental sample outside of the human host (McLean et al., 2013). An automated platform was used to generate genomic DNA by the multiple displacement amplification (MDA) technique from hundreds of single cells in parallel. Thus, the bacterial culture or DNA source of the genomic sequence obtained through MDA cannot be made available (Information source: http://www.ncbi.nlm.nih.gov/biosample/SAMN02436407, also see reference (McLean et al., 2013).

#### Strains sequenced by HMP

A total of six strains (F0185, F0566, F0568, F0569, F0570, and W4087) were sequenced by The Genome Institute of Washington University collaborated with the Data Analysis and Coordination Center (DACC) of the Human Microbiome Project (HMP) and the Human Oral Microbiome Database and were funded by a consortium of institutes including the National Human Genome Research Institute (NHGRI)/National Institutes of Health (NIH), and the National Institute of Dental and Craniofacial Research (NIDCR). Strain F0568 and F0569 were isolated in the 1980s in the USA from the subgingival plaque biofilm of black, non-Hispanic male subjects (53 and 39 years old respectively) diagnosed with moderate periodontitis. F0570 was isolated in the 1980s in the USA from a 39 years old non-Hispanic white male diagnosed with moderate periodontitis. Strain F0185, F0566, and W4087 were reported to be isolated from the oral cavity/mouth of human subjects. Information source: GenBank records in Table 1.

#### SJD2

This strain was isolated from subgingival plaque of a patient in China with chronic periodontitis. It was shown to have high virulent properties comparable with those of the strain W83 in a mouse abscess model (Liu et al., 2014). It was reported to have a higher number of SJD2-specific genes which suggests that strains isolated from a periodontal pocket of Chinese patients with chronic periodontitis may have distinct genes (Liu et al., 2014).

#### HG66

HG66 (also known as DSM 28984) was isolated in Roland R. Arnold's laboratory at the Emory School of Dentistry, Atlanta, GA in the 1960s and was maintained in Jan Potempa's laboratory since 1989. This strain was of interest because it does not retain gingipains on the cell surface, instead releases the majority of proteases in a soluble form. In fact HG66 secretes all carboxy terminal domain-bearing proteins as soluble substances. Information source: http://www.ncbi.nlm.nih.gov/biosample/SAMN02732406 and Siddiqui et al. (2014).

#### A7436

This strain was isolated from the subgingival plaque of the tooth abscess of a refractory periodontitis patient by V.R. Dowell, Jr., at the Centers for Disease Control and Prevention in Atlanta, GA, in the mid-1980s. Information source: http://www.ncbi.nlm.nih.gov/biosample/SAMN03366764 and Chastain-Gross et al. (2015)

#### AJW4

This strain was isolated from the subgingival plaque of the tooth abscess of a periodontitis patient by R.J. Genco and colleagues in 1988 at SUNY-Buffalo, and described by A. Progulske-Fox and colleagues as a minimally invasive strain during *in vitro* cell culture studies. Information source: http://www.ncbi.nlm.nih.gov/biosample/SAMN03372093.

#### Ando

This strain was isolated from the gingival sulcus of a human oral cavity in Japan in 1985. The genome of this strain was sequenced because it was reported to express a 53-kDa-type Mfa1 fimbrium, a major fimbrilin variant of Mfa1 previously known in many *P. gingivalis* strains. Information source: http://www.ncbi.nlm.nih.gov/biosample/?term=SAMD00040429 and Nagano et al. (2015), Goto et al. (2015).

#### 381

Strain 381 was isolated from the subgingival plaque of the tooth abscess of a localized chronic periodontitis patient by S. Socransky, A. Tanner, A. Crawford and colleagues at the Forsyth Dental Center (currently The Forsyth Institute), in the early 1970s. Information source: http://www.ncbi.nlm.nih.gov/biosample/SAMN03656156 and Chastain-Gross et al. (2017).

#### A7A1-28

A strain isolated from subgingival plaque of the tooth abscess of a periodontitis patient, with non-insulin dependent diabetes mellitus, by M.E. Neiders and colleagues in the mid-1987 at SUNY-Buffalo, and was described as a virulent strain with atypical fimbriae and capsule phenotypes. Information source: http://www.ncbi.nlm.nih.gov/biosample/SAMN03653671.

#### MP4-504

This strain is a low-passage (fewer than five passages) clinical isolate sampled from the periodontal pocket (8 mm probing depth) of a chronic periodontitis patient at the University of Washington Graduate Periodontics Clinic in 1991. The important characteristics of this strain include stable adherence to oral streptococci, enhanced invasion of gingival epithelial cells (GECs), strong inhibition of IL-8 production by GECs, and the ability to transfer DNA by conjugation at high efficiencies (To et al., 2016).

### Data analysis

#### 16S rRNA phylogeny

For the 16S rRNA gene phylogeny, *16S rRNA* gene sequences were extracted from the genomes of the 19 *P. gingivalis* strains based on NCBI's annotation (the ^*^genomic.gff file in each of the downloaded genome folder). Sequences were pre-aligned with MAFFT v6.935b (2012/08/21) (Katoh and Standley, 2013) and leading and trailing sequences not present in all sequences were trimmed. The trimmed and aligned sequences, with an alignment length of 1,425 bases and representing 20 unique sequences, were subjected to QuickTree V 1.1 (Howe et al., 2002) using the “-kimura” option to calculate the substitution rate. A copy of the *16S rRNA* gene sequence from *Porphyromonas asaccharolytica* (PaDSM20707) was used as the out-group during the phylogenetic tree construction.

#### Core and unique proteins

To study the phylogenetic relationship based on more genes/proteins, protein sequences annotated by NCBI were used. Together with the outgroup species PaDSM20707, a total of 41,625 proteins were annotated by NCBI, including 39,926 from the 19 *P. gingivalis* genomes and 1,699 from PaDSM20707. Of the 39,926 *P. gingivalis* proteins, 37,667 are ≥ 50 amino acids in length and were searched for homologous clusters using the “blastclust” software V.2.2.25 (http://www.ncbi.nlm.nih.gov/Web/Newsltr/Spring04/blastlab.html). Various sequence identity cutoffs ranging from 10 to 95% and two minimal alignment length cutoffs 50 and 90% were used for identifying the protein clusters. Proteins in each set of the identified clusters were aligned with MAFFT and poorly aligned regions were filtered by Gblocks 0.91b (Talavera and Castresana, 2007). Trees were constructed with FastTree 2.1.9 (Price et al., 2010) using the JTT protein mutation model (Jones et al., 1992) and CAT+–gemma options to account for the different rates of evolution at different sites. The reliability of tree splits were reported as “local support values” based on the Shimodaira-Hasegawa test (Shimodaira and Hasegawa, 2001). For comparison, all 41,625 proteins were also subject to the PhyloPhlAn software (Segata et al., 2013) version 0.99 (8 May 2013).

To identify proteins that are unique for each genome, all the 39,926 *P. gingivalis* proteins were searched against each other using BLASTP 2.2.25 with default parameters (Altschul et al., 1997). Those that did not match any other protein with expected *e* value ≤ 10 were considered unique among the 19 genomes.

#### Whole genome nucleotide comparisons

Pairwise whole genome nucleotide to nucleotide sequence alignment were plotted using NUCmer (NUCleotide MUMmer) version 3.1 (Delcher et al., 2002). To compare the whole genome DNA similarity by the oligonucleotide frequency, all possible 20-mer sequences present in the 20 genomes, including that of *P. asaccharolytica* strain DSM 20707 used as an out-group, were categorized and the number of genomes in which a 20-mer was present was recorded. Any given oligonucleotide can have a maximum of 20 (i.e., present in all 20 genomes) and a minimum of 1 (unique, found in only a single genome). To plot the oligonucleotide frequencies, an overall frequency for every 500 bases across the entire genome was calculated by recording the total number of genomes that all the possible 20-mer in the 500 bases can be found in (maximal 20, minimal 1). Each of the 500 bases windows was colored based on the genome frequency. Another plot was created similarly except that the non-coding regions were masked with light blue color to highlight the oligonucleotide frequencies for the areas that correspond to both forward (upper) and reverse-complement (lower) protein coding sequences.

#### Comparative functional genomics

Three functional annotation systems were used and compared in this study for all the 20 genomes– (1) the NCBI prokaryotic genome annotation pipeline (Tatusova et al., 2016), (2) the SEED and RAST (Rapid Annotation using Subsystem Technology) (Overbeek et al., 2014), and (3) the KOALA (KEGG Orthology And Links Annotation) (Kanehisa et al., 2016). The NCBI annotation results were downloaded from the NCBI FTP site described in the Sequence Sources above. The genomic DNA sequences were sent to the SEED server (Aziz et al., 2012) using the Linux command-line and network-based SEED API downloaded from the SEED server web site (http://blog.theseed.org/servers/installation/distribution-of-the-seed-server-packages.html). The NCBI annotated proteins were sent to the BLastKoala website (http://www.kegg.jp/blastkoala) to identify the KEGG Orthologs. The results of both NCBI and RAST annotations were compared by several text based keyword searches. To identify more proteins in a particular functional category that were somehow annotated in certain genomes but not in others, protein sequences that were annotated in the same category from all 20 genomes were collected and used as the query to search for more proteins of the same functional category. NCBI BLASTP was used for this purpose and proteins with ≥ 95% sequence identity to and ≥ 95% coverage of the query sequences were identified as highly similar proteins. The number of proteins related to the IS5 transposase family was identified by the BlastKOALA program (Kanehisa et al., 2016) with the matching to the KEGG Orthology (KO) number K07481. Additional functional comparison results were also made available as several files in Excel format.

### Data and results availability

To facilitate further comparison and future studies, all the data and results generated in this study, including the original downloaded sequences, annotations, the comparative results presented in this paper, as well as additional complete results that were not mentioned or discussed, are available for download from this FTP data repository site: ftp://www.homd.org/publication_data/20160425.

## Results and discussion

### Summary of genome annotations

The first *P. gingivalis* genome released was that of the strain W83 in 2003 and the latest one was released in February 2016. Of the 19 genomes, eight were assembled into a single contig and were considered complete and finished genomes; the remaining were released as various numbers of sequence contigs assembled from whole genome shotgun (WGS) sequence reads. The sequence of JCVI SC001 appears to have a 1-contig circular sequence under the Genbank Accession number CM001843, however it is a pseudo-contig generated by ordering the 284 unassembled contigs (accession number APMB01000000) based on the homologous matches to the genome of TDC60 (McLean et al., 2013) and joining the ordered contigs with 282 100-N spacer sequences (total N length is 28,200 bps). Thus, it is not considered a complete or finished genome. Examining the sequences for the presence of Ns reveals the “completeness” of the genomes. Table 2 shows the reported length, non-N length, total number of Ns and the distribution of the N fragments in the genomic sequences. Overall strain A7A1-28 is the smallest of the completed *P. gingivalis* genomes with a size of 2,249,024 bps. HG66 has the largest size of all the sequenced *P. gingivalis* genomes at 2,441,680 bps after removing the 100 Ns placed at the end of the sequence. The placement of the 100 Ns at the end of the sequence was due to the unsuccessful attempt to circularize the sequence with the minimus2 software used by the PacBio sequencer at default settings (personal communication). For this reason the HG66 genome should not be considered complete. Almost all the unfinished draft genomes consist of various numbers of Ns ranging from 698 Ns in SDJ2 to 7,200 Ns in F0569 (Table 2). It is likely that some of these published contigs were assembled based on a reference genome and the Ns had been filled in the gaps. Hence the true order of genes identified by the annotation process may not be correct.

**Table 2:** **Effective (non-Ns) sizes of the genomes**.

**Strain**	**Contigs**	**Size(bps)**	**Non-N size(bps)^a^**	**Ns (bps)**	**N fragment size range (fragment count)**
HG66	1	2,441,780	2,441,680	100	100 (1)
JCVI_SC001	1	2,426,396	2,398,196	28,200	100 (282)
381	1	2,378,872	2,378,872	0	None
MP4-504	92	2,373,453	2,373,453	0	None
AJW4	1	2,372,492	2,372,492	0	None
A7436	1	2,367,029	2,367,029	0	None
ATCC_33277	1	2,354,886	2,354,886	0	None
W83	1	2,343,476	2,343,476	0	None
TDC60	1	2,339,898	2,339,897	1	1 (1)
SJD2	117	2,329,548	2,328,850	698	4–256 (23)
F0568	154	2,334,744	2,328,244	6,500	100 (65)
F0566	192	2,306,092	2,300,992	5,100	100 (51)
F0570	117	2,282,791	2,278,391	4,400	100 (44)
A7A1-28	1	2,249,024	2,249,024	0	None
W50	104	2,242,062	2,242,060	2	1 (2)
F0569	111	2,249,227	2,242,027	7,200	100 (72)
F0185	113	2,246,368	2,240,268	6,100	100 (61)
Ando	112	2,229,994	2,227,972	2,022	10–100 (61)
W4087	114	2,216,597	2,212,597	4,000	100 (40)

a *Genomes are ordered based on the non-N size*.

Table 3 gives a numeric summary of the genome annotation results by the NCBI Prokaryotic Genome Annotation Pipeline (released 2013, http://www.ncbi.nlm.nih.gov/genome/annotation_prok/). The NCBI pipeline is capable of identifying more than just the protein-coding genes, rRNAs and tRNAs, including several interesting types of genes such as binding sites, repeat sequences, pseudo-genes, and several types of non-coding RNAs (ncRNAs). However, since the NCBI pipeline is quite new, more features are still being added and since some of the annotations of these *P. gingivalis* genomes were done prior to 2013, the annotation results may not be comprehensive until the annotation is updated again based on the latest NCBI pipeline.

**Table 3:** **Summary of the NCBI annotation^a^^,^^b^**.

**Strain**	**Protein coding**	**tRNA**	**rRNA**	**tmRNA^d^**	**Repeat region**	**Binding site**	**Pseudo-gene**	**ncRNA^c^**				**Other**	**Annotation Release Date^e^**
								**Antisense-RNA**	**RNase-P-RNA**	**Auto-catalytically spliced intron**	**Other ncRNA**		
5W83	1,909	53	12	0	0	0	0	0	0	0	0	41	2014-01-31
ATCC_33277	2,090	53	12	0	210	0	0	0	0	0	0	0	2011-11-26
TDC60	2,220	53	12	1	380	7	0	0	0	0	1	34	2011-08-17
W50	2,016	48	3	1	0	8	0	1	1	0	0	0	2012-06-25
JCVI_SC001	2,354	45	3	1	0	8	0	1	1	0	0	0	2013-04-23
F0568	2,410	46	3	1	0	7	0	0	1	0	0	0	2013-09-16
F0569	2,297	46	3	1	0	7	0	0	1	1	0	0	2013-09-16
F0570	2,315	44	3	1	0	7	0	0	1	1	0	0	2013-09-16
F0185	2,233	45	3	1	0	7	0	0	1	0	0	0	2013-09-16
F0566	2,392	45	3	1	0	7	0	0	1	1	0	0	2013-09-16
W4087	2,202	45	3	1	0	7	0	0	1	1	0	0	2013-09-16
SJD2	2,012	48	3	0	3	0	62	0	0	0	0	0	2013-12-04
HG66	1,958	53	12	0	3	5	38	0	1	0	0	0	2014-10-22
A7436	2,004	53	12	1	4	0	3	0	1	0	0	0	2015-08-11
AJW4	2,002	53	12	1	2	0	2	0	1	0	0	0	2015-08-26
Ando	1,770	47	4	0	0	0	0	0	0	0	0	0	2015-11-27
381	1,968	53	12	1	3	0	9	0	1	1	0	0	2015-10-14
A7A1-28	1,841	53	12	1	5	0	37	0	1	0	0	0	2015-11-17
MP4-504	1,889	47	3	0	3	2	99	0	1	0	0	0	2016-02-09

a *Data analyzed based on the gff files of each genome generated by the NCBI annotation pipeline*.

b *Detail information provided by NCBI can also be downloaded from ftp://www.homd.org/publication_data/20160425/1_Sequence_Sources/*.

c *Mon-coding RNA*.

d *Trans-messenger RNA: a bacterial RNA molecule with dual tRNA-like and mRNA-like properties*.

e *NCBI annotation release dates were based on the dates reported in the protein.gbff file in the above FTP link*.

In addition to the NCBI annotations, RAST (Rapid Annotations using Subsystems Technology) is also a popular pipeline for annotating microbial genomes (Aziz et al., 2008). All the 19 *P. gingivalis* genomes, as well as the chosen outgroup *P. asaccharolytica* DSM20707 were subjected to the RAST pipeline and the results were compared with those done by the NCBI pipeline. As shown in Table 4, both the RAST and NCBI pipelines identified almost the same number of rRNA and tRNA genes. However, the numbers of protein-coding genes varied quite significantly between the two pipelines. Although most of the genes were commonly identified, up to hundreds of protein-coding sequences can be missed by either system. Moreover, 86% (6,422 of 7,382 for all the 19 genomes) of these uniquely identified genes code for hypothetical proteins and 80% are shorter than 100 amino acids in length (in fact, only 94 have lengths ≥ 500 amino acids), thus the impact due to the annotation discrepancy may not be as significant especially when drawing conclusions in genome-wide systematic analysis or metabolic pathway capability.

**Table 4:** **Comparison of NCBI and RAST genome annotations^a^**.

**Strain**	**Total NCBI**	**Total RAST**	**Common/Unique^b^**	**5S rRNA**	**16S rRNA**	**23S rRNA**	**tRNA**
W83	1,909	2,163	1784/80/334	4	4	4	53
ATCC_33277	2,090	2,092	1911/154/144	4	4	4	53
TDC60	2,220	2,090	1880/286/167	4	4	4	53
W50	2,016	2,036	1887/102/123	1	1	1	48
JCVI_ SC001	2,354	2,136	2030/276/78	1	1	1	45/42
F0568	2,417	2,096	1939/403/111	1	1	1	46
F0569	2,297	1,982	1845/377/92	1	1	1	46
F0570	2,316	2,063	1912/338/107	1	1	1	44
F0185	2,236	2,005	1862/319/107	1	1	1	45
F0566	2,395	2,044	1885/428/112	1	1	1	45
W4087	2,204	1,973	1850/303/92	1	1	1	45
SJD2	2,020	2,166	1845/136/271	1	1	1	48/47
HG66	1,958	2,215	1881/58/298	4	4	4	53
A7436	2,004	2,173	1898/84/239	4	4	4	53
AJW4	2,002	2,139	1884/104/226	4	4	4	53
Ando	1,788	1,989	1674/76/275	2	1	1	47
381	1,968	2,108	1853/91/221	4	4	4	53
A7A1-28	1,841	2,039	1736/89/269	4	4	4	53
MP4-504	1,891	2,181	1806/68/347	1	1	1	47

a *Only protein-coding, rRNA and tRNA genes were compared since these are the only types of genes annotated by RAST*.

b The three numbers shown (X/Y/Z) are X, common genes, genes with ≥ 80% overlapped based on the annotated start and end postion; Y, RAST unique genes, gene annotated by RAST without overlap of any NCBI gene; Z, NCBI unique genes, genes annotated by NCBI without overlap to any RAST gene. There are genes that are partially overlapping to each other with < 80% of the length not included.

A list of the 960 (7,382–6,422) non-hypothetical proteins is provided at the link (ftp://www.homd.org/publication_data/20160425/2_Summary_of_Genome_Annotations/Non-overlap_Non-hypothetical_protein_identified_by_NCBI_or_RAST.fasta).

### 16S rRNA phylogeny

The 16S rRNA sequences have been used to infer the evolutionary relatedness of the prokaryotes due to its slow rate of evolution (Woese et al., 1990). However, multiple *rRNA* genes including 16S rRNAs are common in prokaryotic genomes (Klappenbach et al., 2000) and the genomic copy number of 16S rRNA varies greatly among species from 1 to 15 (Vetrovsky and Baldrian, 2013). The number of rRNA genes was reported to correlate with the rate at which phylogenetically diverse bacteria respond to resource availability (Klappenbach et al., 2000). As shown in Table 4, all of the eight genomes which had been assembled to a single contig contain four copies of 5S, 16S, and 23S rRNA genes respectively, thus it is reasonable to believe that all P. gingivalis genomes have four copies of the rRNA operons. The lower number of rRNA genes in the unfinished genomes is likely due to the incompleteness of the sequences and is also likely due to the fact that genomes sequenced by short reads sequencing platforms such as those of the Illumina sequencers cannot be easily assembled across the repeated regions such as the highly conserved rRNA operons.

**Figure 1:**
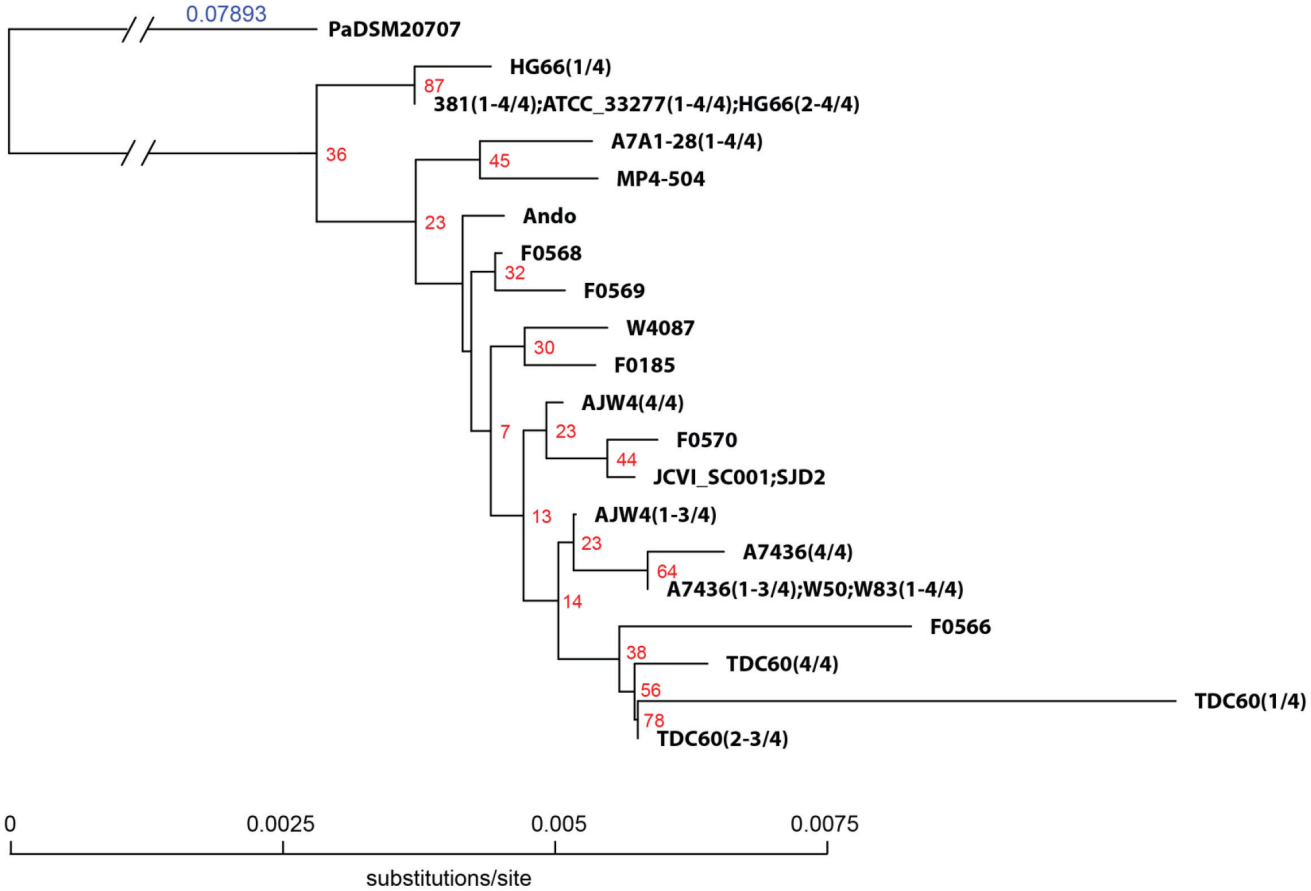
**Phylogenetic tree of *P. gingivalis 16S rRNA* gene sequences**. A total of 24 unique *16S rRNA* gene sequences were extracted from the genomes of 19 *P. gingivalis* strains annotated by NCBI. Sequences were pre-aligned with MAFFT v6.935b (2012/08/21) (Katoh and Standley, 2013) and leading and trailing sequences not present in all sequences were trimmed. The trimmed aligned sequences represent 20 unique sequences and were subject to QuickTree V 1.1 (Howe et al., 2002) using the “-kimura” option to calculate the substitution rate. Sequence of *P. asaccharolytica* strain DSM 20707 (PaDSM20707) was used as out-group. The branch length of the out-group was truncated to fit the tree in the figure and the substitution rate is indicated with the blue number. The red numbers next to the branching point are the bootstrap values based on 100 iterations. Sequences of different strains were separated by semicolons and the number of sequences were indicated in the parentheses in the format of (x–y/z), where x and y are the start and end IDs and z the total number in the strain.

The 16S rRNA sequences of all the 19 genomes annotated by NCBI were extracted and aligned for the construction of a phylogenetic tree. Based on the annotation, there are a total of 24 unique 16S rRNA gene sequences identified from the 19 genomes (Table 5, first column), plus the sequence of a close species P. asaccharolytica strain DSM 20707 (Accession Number CP002689), making it a total of 25 unique sequences in the study. However, many of the sequence differences are due to different annotated lengths. After aligning all the 24 (25 if including the outgroup sequence) unique sequences and trimming off the leading and trailing sequences not present in all copies (trimmed aligned length = 1,425 bps), the aligned portion of several sequences are identical and the number of unique sequences was reduced to 20 (second column of Table 5). Strains 381, A7A1-28, ATCC 33277, and W83 all have four copies of identical sequences in their genomes (Table 5, last column). The aligned regions of all the 16S rRNA gene sequences from ATCC 33277, 381, and HG66 have identical sequences, indicating the close evolutional distance of these strains. Strain A7436 shared three of its four copies of 16S rRNA sequences identically with those of W83. Together with the single copy from W50, they formed an identical group of sequences. W50 has been known to be a close strain of W83, thus the identical sequences between these two are not surprising. The explanation of identical copies of the 16S rRNA sequence in the genome is apparently due to the gene duplication event and the fact that several strains shared identical duplicated sequences suggested that the duplication event occurred after the speciation. Strains A7436, AJW4, and HG66 had three strain-specific identical sequences with the 4th copy different from the other three. Overall, all the P. gingivalis 16S rRNA gene sequences were extremely similar and often have only a single number of nucleotide mismatches between any two strains (if not identical). Altogether only 16 loci on the gene had nucleotide variations (some could have variations in more than one strain), with the exception of one copy in TDC60, which had a series of A → C or G → C transversions between position 50 and 130 and two single nucleotide insertions at position 174 and 233. It is thus the most divergent sequence of among all 19 P. gingivalis genomes. These aligned and trimmed sequences, including the outgroup sequence from P. asaccharolytica strain DSM 20707, were used to construct a phylogenetic tree based on Kimura's nucleotide substitution model and the result is shown in Figure 1. The phylogenetic tree depicts a likely evolutionary path for these different P. gingivalis strains. The strains 381, ATCC 33277, and HG66 appeared to be closer to the potential common ancestor, based on the tree topology inferred with a close species as the outgroup sequence. The other strains gradually diversified into deeper branching nodes with two of the sequences from strains F0566 and TDC60 as the most deeply branched and mutated from the common ancestor, which was inferred by using the sequence of a neighboring species (PaDSM20707) as an outgroup.

**Table 5:** **Unique *16S rRNA* gene sequences in *P. gingivalis* genomes**.

**Original sequence**	**Trimmed sequence^a^**	**Copy number**	**Original length (bps)**	**Strains (copy number)^b^**
Unique Seq 1	Unique Trimmed Seq 1	4	1,422	381 (4)
Unique Seq 2		4	1,475	ATCC33277 (4)
Unique Seq 3		3	1,538	HG66 (3)
Unique Seq 4	Unique Trimmed Seq 2	1	1,538	HG66
Unique Seq 5	Unique Trimmed Seq 3	3	1,422	A7436 (3)
Unique Seq 6		5	1,475	W50; W83 (4)
Unique Seq 7	Unique Trimmed Seq 4	1	1,422	A7436
Unique Seq 8	Unique Trimmed Seq 5	4	1,422	A7A1-28 (4)
Unique Seq 9	Unique Trimmed Seq 6	3	1,422	AJW4 (3)
Unique Seq 10	Unique Trimmed Seq 7	1	1,422	AJW4
Unique Seq 11	Unique Trimmed Seq 8	1	1,521	TDC60
Unique Seq 12		1	1,520	TDC60
Unique Seq 13	Unique Trimmed Seq 9	1	1,522	TDC60
Unique Seq 14	Unique Trimmed Seq 10	1	1,520	TDC60
Unique Seq 15	Unique Trimmed Seq 11	1	1,475	JCVI SC001
Unique Seq 16		1	1,538	SJD2
Unique Seq 17	Unique Trimmed Seq 12	1	1,475	Ando
Unique Seq 18	Unique Trimmed Seq 13	1	1,520	W4087
Unique Seq 19	Unique Trimmed Seq 14	1	1,520	F0569
Unique Seq 20	Unique Trimmed Seq 15	1	1,520	F0568
Unique Seq 21	Unique Trimmed Seq 16	1	1,520	F0185
Unique Seq 22	Unique Trimmed Seq 17	1	1,520	F0566
Unique Seq 23	Unique Trimmed Seq 18	1	1,542	MP4-504
Unique Seq 24	Unique Trimmed Seq 19	1	1,520	F0570
Unique Seq 25^c^	Unique Trimmed Seq 20	2	1,517	PaDSM20707 (2)

a *Sequences were pre-aligned with the software MAFFT v6.935b (2012/08/21) (Katoh and Standley, 2013) with default setting; after trimming the leading and trailing sequences not present for all genomes, the trimmed aligned sequence length is 1,425 bps in length*.

b *If multiple copies of identical sequences are present, the copy number is indicated in the parenthesis*.

c *Sequence of P. asaccharolytica strain DSM 20707 (from Genbank ID: CP002689) was included as outgroup*.

### Core and unique proteins

The phylogenetic relationship inferred based on the *16S rRNA* gene sequences reported above can only represent the evolution of this particular gene, hence a gene tree. A more comprehensive way of studying the evolutionary relatedness of different genomes is to use as much genomic information as possible in the analysis (i.e., phylogenomics). A popular approach is to use the core proteins for the construction of a tree that may be closer to a true species tree, if such a tree exists, or if there is no true species tree, may reflect more on the relatedness of these strains at the genomic level. The concept of “core” proteins is ideally defined as proteins that are present and required by all the genomes in study, however the identification of such a group of proteins, namely orthologs, is not straightforward and the results vary depending on the criteria used. It is challenging, if not impossible, to identify all the orthologous proteins among a group of genomes. In general, genomes of closer species or strains share more orthologs; however any percent protein sequence identity chosen as the cutoff to test whether a group of homologous proteins are truly orthologs (or paralogs) can always include some false positive and negative orthologs. Nevertheless, one can still hypothesize that a more reliable evolutionary relationship of a group of genomes can be obtained if the use of higher or lower percent identity constrains does not affect the overall tree topology.

To test this hypothesis, the “core” proteins were first identified among all *P. gingivalis* genomes under different cutoffs. Based on the NCBI annotation, a total of 39,926 protein sequences were identified. However, for some unknown reasons, some of the annotated protein lengths were as short as one or two amino acids. For example, proteins with Genbank IDs GAP82138.1, GAP81676.1, and GAP81848.1 in strain Ando were identified with only 1, 2, and 2 amino acids in length respectively. These clearly were annotation errors caused by the computational bugs in the annotation pipeline. In this analysis, only protein sequences with a minimal length of 50 amino acids were used (a total of 37,667 proteins) for identifying core proteins. They were subject to the “blastclust” program (http://www.ncbi.nlm.nih.gov/Web/Newsltr/Spring04/blastlab.html) to identify clusters of proteins that share a certain degree of sequence homology and with specified alignment length coverage. In this analysis, if a protein is present (i.e., meets the % identity and alignment cutoffs specified) in all 19 genomes (or 20 genomes if PaDSM20707 was used as the outgroup in some results) it is considered as a core/shared protein, and if a protein is only present in a single genome it is considered as a strain-specific unique protein.

**Figure 2:**
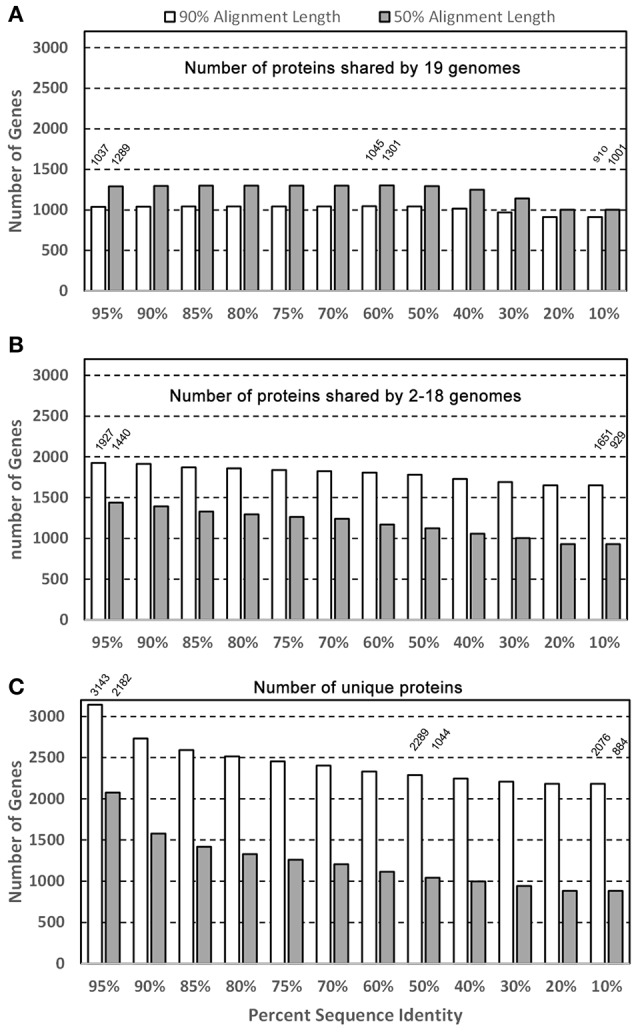
**Core and unique genes in *P*. *gingivalis* surveyed by sequence identity and alignment length**. Of the 39,926 NCBI annotated *P. gingivalis* proteins, 37,667 are ≥ 50 amino acids in length and were searched for homologous clusters using the “blastclust” software V.2.2.25 (http://www.ncbi.nlm.nih.gov/Web/Newsltr/Spring04/blastlab.html). Various sequence identity cutoffs ranging from 10 to 95% and two minimal alignment length cutoffs 50 and 90% were used as the program parameters to identify the protein clusters in the three categories **(A)** clusters containing proteins from all 19 genomes; **(B)** clusters containing proteins from 2 to 18 genomes; and **(C)** clusters with protein from only 1 genome.

Figure 2 shows the potential numbers of both core and unique proteins in the 19 genomes analyzed with “blastclust” by varying two parameters: Sequence percent identity cutoffs (from 95 to 10%) and percent alignment length (90 and 50%). Figure 2A shows that regardless of sequence identity cutoffs; the number of core proteins stays relatively constant around 1,000 with 90% as the alignment length cutoff. The number of core protein groups increased gradually from 1,037 at 95% identity, maximized at 1,045 at 60%, then decreased to 910 at 10%. The reason for the increase from 95 to 60% was due to more core protein groups clustered together at lower % identity. The decrease after 60% identity was due to the fact that different protein groups identified with identity ≥ 60% began to merge into fewer groups. The 1,037 shared proteins were detected under most stringent conditions thus it is reasonable to state that at least 1,037 core proteins were detected based on 19 strains. This number is expectedly smaller than the 1,476 detected in the core genome based on eight *P. gingivalis* strains (Brunner et al., 2010). It should also be noted that the core genes/proteins are not the same as the “essential” genes, for which only ca. 400 were experimentally detected previously (Klein et al., 2012).

For the purpose of identifying a core/shared set of proteins for constructing a phylogenomic tree, the 1,045 core proteins identified at 60% sequence identity and 90% alignment length cutoffs were used for sequence alignment and tree building. This set of sequences is available for download in the data repository FTP site mentioned in the Material and Methods. In addition, as expected when the percent alignment length was decreased from 90 to 50%, more proteins were identified as core proteins, e.g., from 1,289 at 95% identity cutoff to 1,301 at 60%, due to the fact that more proteins share the same percent identity over shorter sequence length.

Figure 2B shows the number of protein groups that are shared by 2 to 18 genomes (partially shared proteins). The number decreases with the lower % identity because similar protein groups that were identified as separate groups merged into a single (but larger) group due to the more relaxed (lower) % identity (e.g., from 1,927 at 95% to 1,651 at 10%). However, contrary to the core proteins above, which require proteins present in all 19 genomes, when the percent alignment decreased, fewer partially-shared proteins were identified. This is as expected because when the percent alignment cutoff was lowered, a protein group which consists of only members from for example 18 genomes, at higher cutoff, now may find a member in the 19th genome thus disqualifying it as the 18-genome partially-shared group.

Figure 2C shows the number of strain-specific proteins that are present in only one genome with a single copy. Similar to the partially-shared groups, as the % identity decreases the number of unique proteins becomes smaller because more proteins from different genomes were lumped together as a homologous group under a lower % identity, resulting in the loss of the “uniqueness”. In addition, for example, at 60% sequence identity and 90% alignment cutoffs, there were 2,289 proteins identified as present in a single genome, but the number was reduced to 1,044 at 50% alignment cutoff–1,245 proteins lost their uniqueness due to the presence of more “similar” proteins found in other genomes.

**Figure 3:**
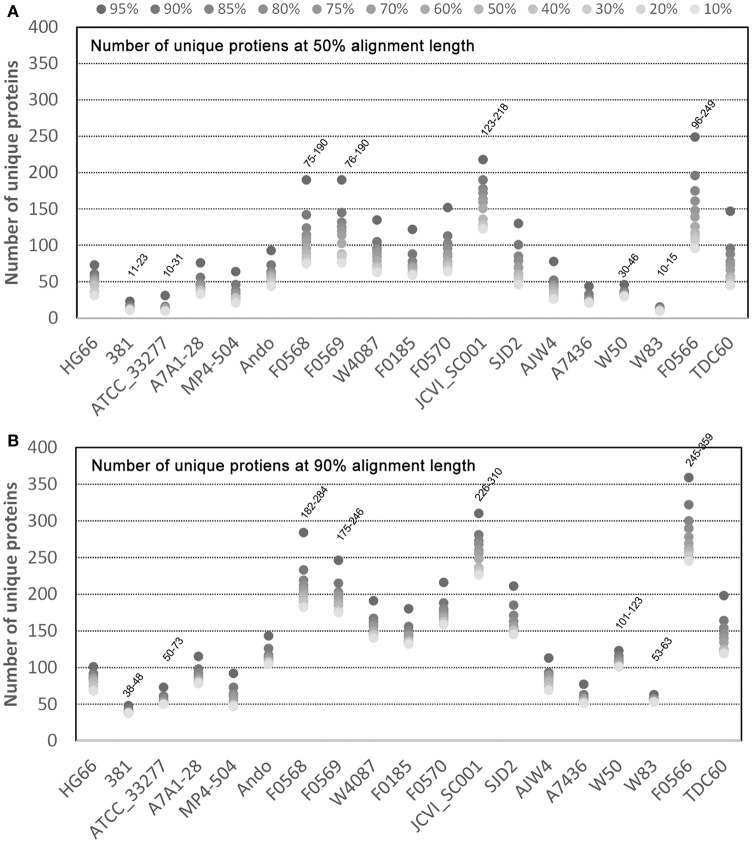
**Unique proteins in 19 *P. gingivalis* strains**. Of the 39,926 NCBI annotated *P. gingivalis* proteins, 37,667 are ≥ 50 amino acids in length and were searched for homologous clusters using the “blastclust” software V.2.2.25 (http://www.ncbi.nlm.nih.gov/Web/Newsltr/Spring04/blastlab.html). Unique proteins of each of the 19 *P. gingivalis* genomes were identified as proteins found in only one genome without any similar counterpart in any other. The total number of clusters that contain only unique proteins for each genome were plotted. Various sequence identity cutoffs ranging from 10 to 95% (dots with varying grayscale color intensity) and two minimal alignment length cutoffs 50% **(A)** and 90% **(B)** were used as the program parameters.

For the unique proteins identified, it would be interesting to observe their distribution in the 19 genomes and the result may help understand which genomes possess more or fewer unique proteins. Figure 3 shows the distribution of the 1,044 unique proteins identified with the 50% alignment cutoff (Figure 3A) and 2,289 with 90% cutoff (Figure 3B). Regardless of the sequence identity and percent alignment cutoff, the results show that some strains possess significantly more unique proteins than others. Four strains, F0566, F0568, F0569, and JCVI SC001 have a significantly higher number of unique proteins under all identification conditions - as high as 96–249 unique proteins (for percent identity 10–95% at 50% alignment length) in the case of F0566 (Figure 3A). On the other hand, strains 381, ATCC 33277, A7436, and W83 are the four strains with the lowest number of unique proteins, only 10–15 unique proteins (10–95% identity; 50% alignment) in the case of W83 (Figure 3A). Interestingly, strain W50, the closest strain to W83, encodes more unique proteins (30–46) even though it is an unfinished draft genome. Apparently the incompletes of the draft genomes are not the cause for the difference in the number of unique proteins (JCVI SC001 and all the F strains are draft genomes). This further suggests that the gaps in the draft genomes may contain only repeated sequences that either do not encode for proteins, or encode repeated proteins that do not contribute much to the genome's uniqueness. However, we cannot rule out the possibility that these regions could still have some unique proteins which would drive up the number already observed.

Another noteworthy observation is that there is a consistent gap between the data points 95% and 90% identity when searching for unique proteins in all strains under both alignment conditions (Figure 3). This suggests that the more proteins identified as unique at 95% became “similar” at 90%. Hence 90% sequence identity may be an ideal cutoff for differentiating homologs and unique proteins, at least at the strain level.

Table 6 lists the percentage of proteins that were annotated in NCBI as the hypothetical proteins (functionally unknown) and the percentage of the unique proteins that were identified with 80% as the sequence identity and 50% alignment length. The total percent hypothetical proteins range from 26% (W50) to as high as 46% (F0566 and F0568), whereas the majority of the unique proteins are hypothetical, from 68% (TDC60) to 100% (W83). Thus, until more functions of the hypothetical proteins are understood, it will still be challenging to understand what each genome's overall “specialty” functions conferred by the unique genes. To give a glimpse of what each genome's most unique functions are, based on the currently available information, Table 7 lists the functional annotations of the non-hypothetical unique proteins for each of the 19 genomes (with default BLASTP parameter, i.e., expected e value ≤ 10) (Altschul et al., 1997). All of them are among the proteins identified above under the most stringent parameters in terms of uniqueness–50% sequence identity and 50% alignment length. Strain JCVI SC001, an environment isolate from a hospital sink drain, has the most diverse functions encoded by these unique proteins. The unique toxin-antitoxin system detected in strain F0569 (a clinical isolate from subgingival plaque biofilm) is also of interest. The toxin-antitoxin system genes when carried on a plasmid is often referred to as the post-segregational killing (PSK) system (Gerdes, 2000) while when carried in the chromosome such a system is involved in stress response and “programmed cell death” (Hayes, 2003). Although indigenous plasmids have never been detected in *P. gingivalis* this does not rule out finding one in the future. Thus, which type of function that this toxin-antitoxin system is involved in can only be speculated at this point of time. Whether these annotations translate to unique functions of the genome, require further investigation to ensure there are no other non-homologous proteins that play similar functions. All of the unique proteins identified are available by strain in the FTP data repository.

**Table 6:** **Percent hypothetical proteins 19 *P. gingivalis* genomes**.

**Strain^a^**	**Total**	**% Total hypothetical(%)**	**Total unique^b^ (80% identity)**	**% Unique hypothetical (80% identity)(%)**
HG66	1,958	28	53	81
381	1,968	27	13	85
ATCC_33277	2,090	42	14	79
A7A1-28	1,841	28	46	78
MP4-504	1,891	27	34	85
Ando	1,788	29	61	70
F0568	2,417	46	114	88
F0569	2,297	45	125	86
W4087	2,204	43	94	78
F0185	2,236	43	72	88
F0570	2,316	44	96	90
JCVI_ SC001	2,354	30	172	72
SJD2	2,020	35	79	82
AJW4	2,002	29	45	76
A7436	2,004	28	25	80
W50	2,016	26	34	91
W83	1,909	35	13	100
F0566	2,395	46	161	86
TDC60	2,220	41	78	68

a *The strains were ordered somewhat according to the 16S rRNA phylogenetic tree shown in Figure 1*.

b *The unique proteins were identified by “blastclust” program with parameters 80% as the sequence identity and 50% alignment length*.

**Table 7:** **Non-hypothetical unique^a^ proteins in 19 *P. gingivalis* genomes**.

**Strain**	**Annotation**
HG66	Glyoxalase
A7A1-28	Beta-galactosidase; putative hydrolase or acyltransferase of alpha/beta superfamily
Ando	DNA polymerase III subunits gamma and tau, partial external scaffolding protein D replication-associated protein A major spike protein G
F0568	DGQHR domain protein
F0569	Toxin-antitoxin system, toxin component, Fic domain protein
W4087	CAAX amino terminal protease family protein phage portal protein, SPP1 family phage uncharacterized protein
F0185	Peptidase S24-like protein
JCVI_SC001	Thioesterase family protein, partial starch-binding protein, SusD-like domain protein, partial spermine/spermidine synthase, partial phage portal protein, lambda family, partial head to-tail joining protein W serine carboxypeptidase domain protein, partial NYN domain protein imidazoleglycerol-phosphate dehydratase domain protein, partial carbohydrate kinase, PfkB domain protein PF13785 domain protein, partial DNA-binding helix-turn-helix protein
SJD2	Transposase ISPsy14
AJW4	Geranylgeranyl pyrophosphate synthase T5orf172 domain-containing protein
A7436	Transposase
W50	Transposase, mutator-like family protein
TDC60	Terminase

a *These proteins were searched against all the proteins in the 19 genomes and matched none but itself at the default BLASTP 2.2.25 parameter (i.e., with expected e value ≤ 10) (Altschul et al., 1997)*.

### Phylogenomics by homologous proteins

**Figure 4:**
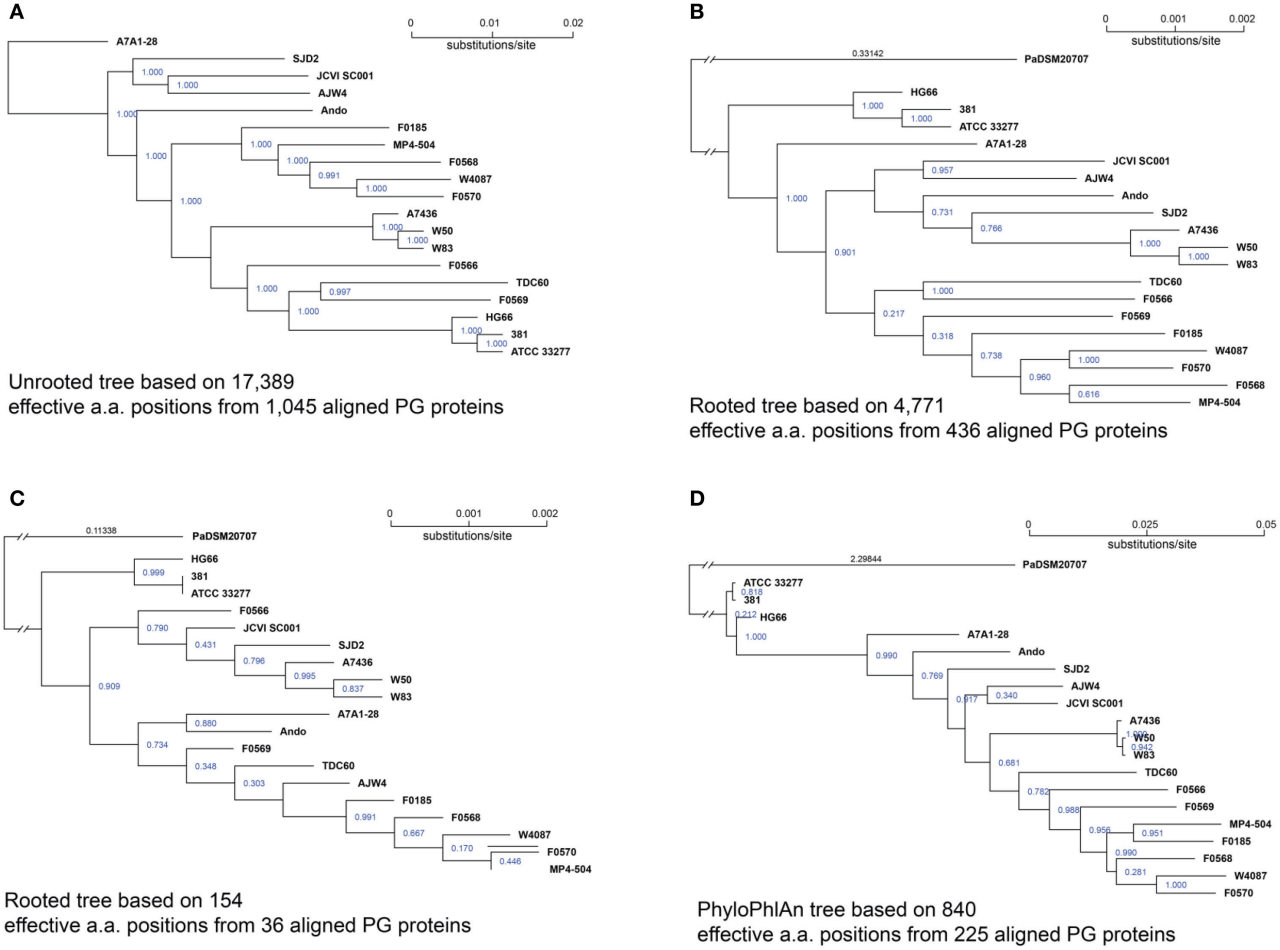
***P. gingivalis* phylogenomic trees based on core proteins identified at various percent sequence identities**. Of the 39,926 NCBI annotated *P. gingivalis* proteins, 37,667 are ≥ 50 amino acids in length and were searched for homologous clusters using the “blastclust” software V.2.2.25 (http://www.ncbi.nlm.nih.gov/Web/Newsltr/Spring04/blastlab.html). **(A)** unrooted tree based on the 1,045 shared proteins identified by “blastclust” with 60% as the sequence identity and 90% as the alignment length cutoffs; the alignment generated a total of 17,389 effective (non-identical) protein sequence positions across all 19 genomes and the tree was constructed based on these positions; **(B)** rooted tree based on 436 proteins (out of 1,045) that are also found in *P. asaccharolytica* strain DSM 20707 (PaDSM20707) with ≥ 50% sequence identity and ≥ 90% alignment length; the alignment generated 4,771 effective protein sequence positions; **(C)** rooted tree based on 36 proteins shared among 20 genomes with ≥ 80% sequence identity and ≥ 90% alignment length. Proteins were aligned with MAFFT v6.935b (2012/08/21) (Katoh and Standley, 2013) and poorly aligned regions were filtered by Gblocks 0.91b (Talavera and Castresana, 2007). Trees were constructed with FastTree 2.1.9 (Price et al., 2010) using the JTT protein mutation model (Jones et al., 1992) and CAT+–gemma options to account for the different rates of evolution at different sites. The reliability of tree splits were reported as “local support values” based on Shimodaira-Hasegawa test (Shimodaira and Hasegawa, 2001) and are printed in blue on the split. The branch length (substitution rate) of the outgroup PaDSM20707 was truncated and the length were printed in black **(B,C)**; **(D)** Rooted tree constructed using PhyloPhlAn (Segata et al., 2013) by directly subjecting all NCBI annotated proteins of the 20 genomes to the software, resulting in 840 effective protein positions from 225 aligned proteins.

Once a group of putative core proteins is identified, they can be concatenated and aligned together and used for compiling a phylogenomic tree to infer a possible evolutionary relationship at a level closer to the species than just any single gene. In this analysis, the 1,045 proteins shared by all 19 genomes at 60% sequence identity and 90% alignment cutoffs (Figure 1A), were first aligned individually with the “mafft” software (Katoh and Standley, 2013). Each of the 1,045 protein sets contained exactly 19 aligned sequences, one from each of the 19 genomes. The aligned proteins were concatenated in the same protein order. This generated a set of 19 mega protein sequences with each consisting of 1,045 concatenated aligned sequences. The poorly aligned sequence regions, including leading and trailing unaligned portions of the sequences, as well as low-confidence parts of the alignment, such as positions that contain many gaps, were removed with the “Gblock” tool (V 0.91) (Talavera and Castresana, 2007). After the Gblock screening, a final set of 19 aligned protein sequences, each with a length of 395,174 amino acids were used for constructing an unrooted tree. However, among the 395,174 aligned amino acids, only 17,389 positions had at least two different amino acids across proteins of all 19 *P. gingivalis* genomes, the remaining 377,785 were all the same amino acids across all genomes. Thus, only those 17,389 informative or effective positions contributed to the pairwise distances calculated among all genomes. Figure 4A is the result of the unrooted tree compiled based on the 1,045 shared proteins processed as described above. The overall topology is quite different from that of the *16S rRNA* tree (Figure 1) with the exception of two very closely related groups of strains, one consists of strains 381, ATCC 33277, and HG66 and another A7436, W50, and W83. This is not surprising because both groups have members with identical *16S rRNA* sequences hence their shared protein sequences are closer to each other in the group than other genomes.

To test whether including proteins from the outgroup species will result in a tree more similar to that of *16S rRNA*, i.e., a tree that is rooted at a potential common ancestor for these strains, ortholog candidates were first identified from the genome of *P. asaccharolytica* DSM 20707, of which the *16S rRNA* sequence was also used for the *16S rRNA* tree. At 90% alignment length cutoff, the number of homologous proteins in *P. asaccharolytica* decreases as the percent sequence identity cutoff increases. The numbers of protein homologous to any of the 1,045 core proteins used for the unrooted tree above are 436, 271, 146, 36, 7, 1, and 0 respectively for percent identity cutoffs 50, 60, 70, 80, 85, 90, and 95%. Figures 4B,C are the two rooted phylogenetic trees constructed based on the 436 (50% identity) and 36 (80% identity) proteins shared between *P. asaccharolytica* DSM 20707 and all 19 *P. gingivalis* strains. After Gblocks screening, the length of the aligned sequences were 12,646 (80% identity) and 177,272 (50% identity) amino acids respectively and the number of effective amino acids positions are 154 and 4,771 respectively. In general, the branch lengths increased with more effective amino acids positions which resulted in greater distances. Again, the only consistent close clusters were the two grouped with identical *16S rRNA*, i.e., the group of 381, ATCC 32277, and HG66, and of A7436, W50, and W83.

Figure 4D is the rooted tree constructed using the software PhyloPhlAn (Segata et al., 2013) version 0.99 (8 May 2013). All 41,625 proteins annotated for the 20 genomes were subject to PhyloPhlAn with the default parameters that excluded proteins shorter than 30 amino acids in length. PhyloPhlAn finds among the input protein matches to a pre-set of the 400 most conserved proteins for extracting the phylogenetic signals. A total of 264 query proteins were matched to the 400 preset core but only 225 were present in all 20 genomes. These proteins were then aligned individually and subsampled based on a sophisticated procedure provided by PhyloPhlAn, which emphasizes regions both universally conserved and phylogenetically discriminating. The final aligned, subsampled, and concatenated sequences had a length of 3,082 aligned amino acids with 840 effective positions. The PhyloPhlAn tree is shown in Figure 4D. Similar to the two rooted trees (Figures 4B,C) and the 16S rRNA tree (Figure 1) the PhyloPhlAn tree also placed the three strains ATCC 33277, 381, and HG66 closest (but much closer) to the root and the remaining strains in a more linearly nested topology.

In summary, the only consensus based on interpretation of the three rooted protein trees and the *16S rRNA* tree is that the group ATCC 33277, 381, and HG66 is less evolved and closest to the common ancestor of this species (inferred based on the distance to the root). Strains W83, W50, and A7436 consistently formed a close group regardless of how the trees were built, but their exact phylogenetic position is inconclusive based on these analyses. Strains F0185, F0568, F0570, W4087, and MP4-504 are also found to be in the proximity of each other, although not as close as the two groups mentioned above. In general more effective/informative aligned amino acid positions resulted in longer branches and pairwise distances. To this end, the unrooted tree (Figure 4A) has the best resolution to reveal the similarity/differences among these strains, in the most genome-wide manner. Until a group of true orthologous proteins are identified (together with the outgroup) a true phylogenetic tree that infers the evolutionary path for this species will not be accessible.

## Comparisons based on whole-genome nucleotide sequences

### NUCmer nucleotide plots

**Figure 5:**
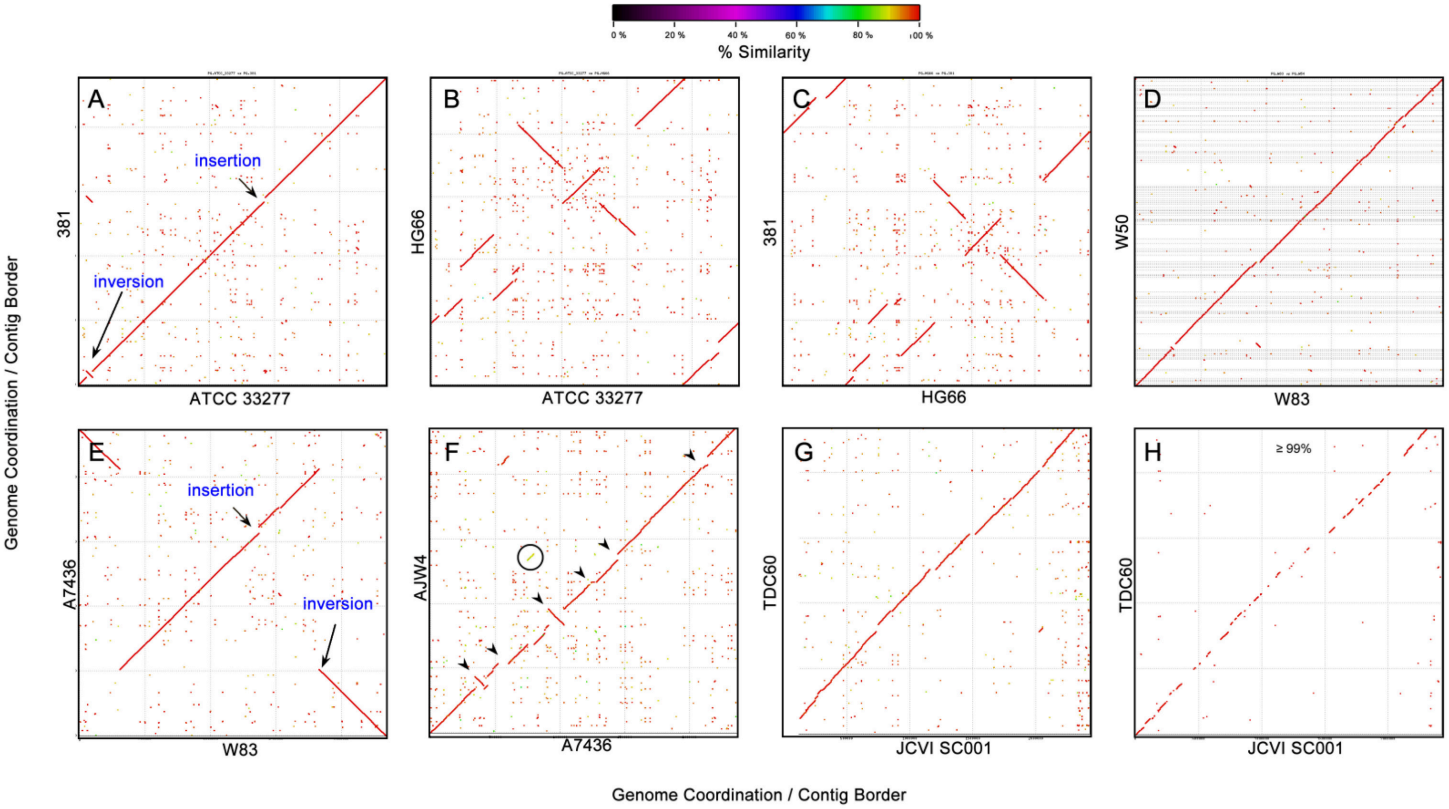
**DNA-DNA sequence alignment between *P. gingivalis* genomes**. Genomic sequence alignment between several pairs of *P. gingivalis* strains were plotted using NUCmer (NUCleotide MUMmer) version 3.1 (Delcher et al., 2002). The sequence percent identities of detected homologous fragments were plotted in gradient colors based on the percentage. The axes are the nucleotide coordination in the genomes. The orders of the contigs in the unfinished genomes were rearranged based on the reference genome (genome on X- axis). **(A)** strain 381 vs. ATCC 33277; **(B)** HG66 vs. ATCC 33277; **(C)** strain 381 vs. HG66; **(D)** W50 vs. W83; **(E)** A7436 vs. W83; **(F)** AJW4 vs. A7436; **(G)** TDC60 vs. JCVI SC001; and **(H)** TDC60 vs. JCVI SC001 showing only the region with percent identity ≥ 99%.

Moving up the scale for comparison, one possible way is the whole genome nucleotide alignment with a commonly used software NUCleotide MUMmer (NUCmer), which identified nucleotide MUMs–minimal unique matches between two genomic sequences (Delcher et al., 2002). Figure 5 shows some of the pairwise alignment results of the 19 *P. gingivalis* genomes. Figure 5A is the nucleotide alignment between strains 381 and ATCC 33277 and the almost perfect diagonal high similarity (red) match line indicates highly similar sequences, with only two visible exceptions – one inversion and one insertion (to 381)/deletion (to ATCC 33277). Interestingly the inverted sequence almost matches the inserted sequence; apparently the inverted sequence was duplicated in the 381 genome and inserted somewhere else in the genome, where the ATCC 33277 genome shows no counterpart. The high DNA sequence similarity between 381 and ATCC 33277 is also supported by the identical *16S rRNA* gene sequence and copy numbers (Figure 1) as well as the protein-based phylogenetic relationships (Figure 4), even though their genomes are not far from identical. The phenomenon that a fairly large chunk of genomic sequence was duplicated and inserted elsewhere in the genome is only observed in strain 381, as evidenced by the NUCmer self-alignment of its genome (data available from the FTP site), but very similar to the alignment between 381 and ATCC 33277). No duplication event was observed in the self-alignment of the other 18 genomes.

Strain HG66 is the genome that is closest to 381 and ATCC 33277 based on *16S rRNA* genes and protein sequences, on the other hand it shows the disconnected high similarity match lines, which indicates more large-scale genomic arrangement between the two close strains – between 381 and HG66 (Figure 5B) and between ATCC 33277 and HG66 (Figure 5C).

The second closest groups of strains are A7436, W50, and W83 and their nucleotide sequences are also highly similar based on the NUMMER plots (Figures 5D,E). However, the contigs of the unfinished draft genome of W50 were rearranged by NUCmer in the order based on the similarity to the W83 sequence. Whether there is a large scale genomic rearrangement between W83 and W50 cannot be known until the genome of W50 is completed. Strain A7436, a finished genome, shows only one inversion of the genome when compared to that of W83 (Figure 5E). The fact that A7436 is not as close to W50 and W83 as the distance between HG66 and 381 (or ATCC 33277) based on *16S rRNA* and protein phylogeny (Figures 1, 4), suggests that the genomes of the group of HG66, 381, and ATCC 33277 have higher genomic sequence rearrangement activity than the A7436-W50-W83 group. The next genome which is closest to the A7436-W50-W83 group is strain AJW4, with several visible (larger fragments) of insertions/deletions and inversions when compared to A7436 (arrows heads in Figure 5F). This relationship is also consistent with the *16S rRNA* gene tree (Figure 1).

Another interesting observation is the alignment between JCVI SC001 and TDC60. These two strains are not among the closest groups based on the *16S rRNA* and protein sequences (Figures 1, 4). The NUCmer plot between these two genomes appears to be a straight diagonal red line (Figure 5G), similar to that between 381 and ATCC 33277. However, since the genomic sequence of JCVI SC001 was not really completed and closed to a circular chromosomal format, the 284 *de novo* assembled contigs were mapped to the genome of TDC60 and the gaps were filled with Ns to form a single pseudo-contig (Genbank Accession CM001843) (McLean et al., 2013). Thus, the contig order in the published single contig genomic sequence of JCVI SC001 may not be correct and the sequence similarity between JCVI SC001 and TDC60 may not be as “straight” as indicated in the NUCmer plot. In fact when the plot was filtered to show only the region with percent identity ≥ 99%, the red line became fragmented with large gaps (Figure 5H), indicating that a large portion of the genomic sequences between these two strains are under 99% similarity.

The complete pair-wise NUCmer plots of the 19 *P. gingivalis* genomes can be viewed in a specifically designed interactive webpage at http://bioinformatics.forsyth.org/publication/20160425. The web page provides interactive tools to choose any two *P. gingivalis* genomes for the NUCmer results, as well as the possibility of viewing the alignment at various percent sequence identity cutoffs.

### Oligonucleotide frequency

**Figure 6:**
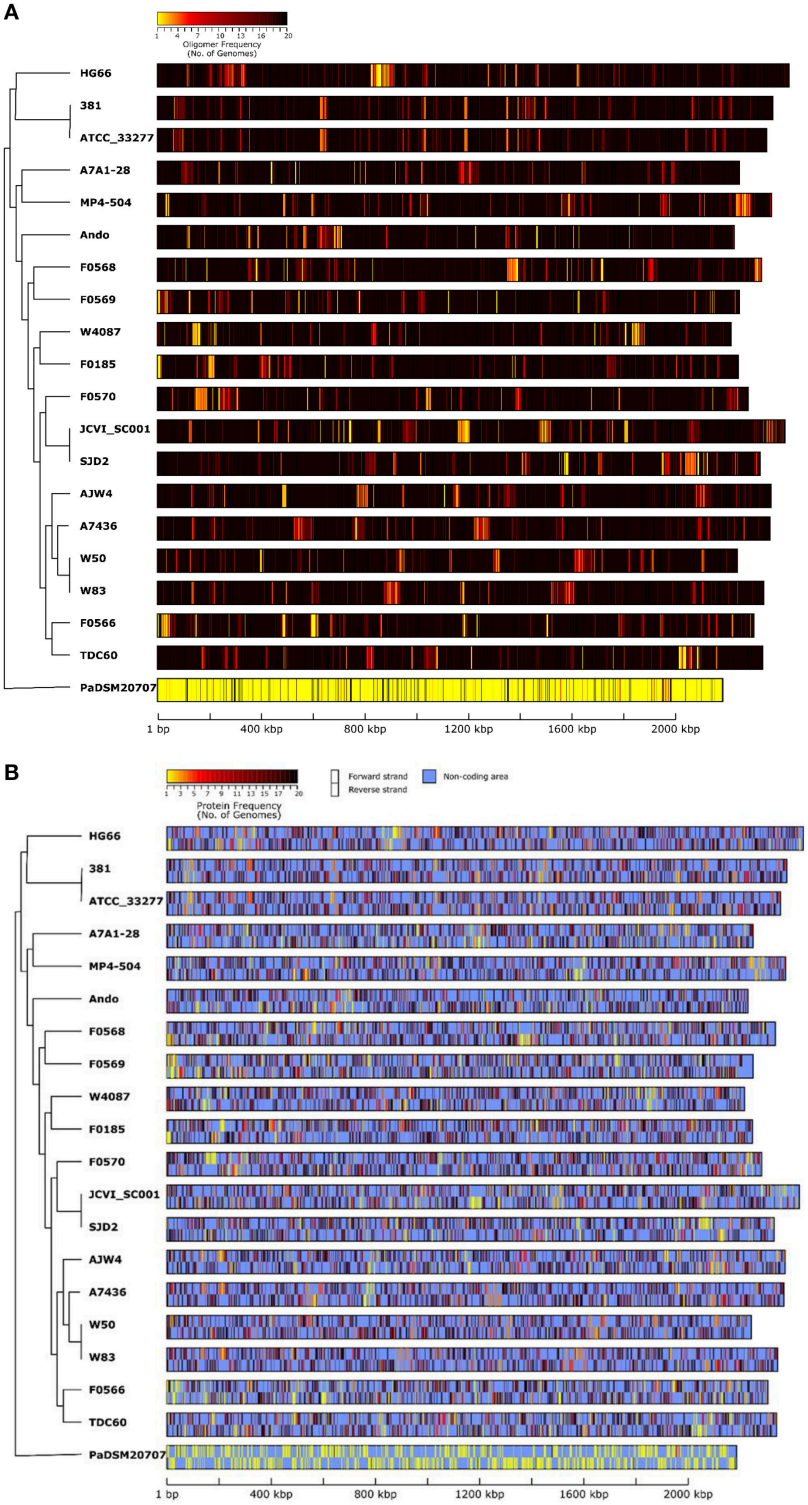
**Genomic DNA similarity of 19 *P. gingivalis* genomes compared by oligonucleotide frequency**. All possible 20-mer sequences present in all genomes, including that of *P. asaccharolytica* strain DSM 20707 (PaDSM2070) used as an out-group, were categorized and the number of genomes in which a 20-mer is present, was recorded. **(A)** was generated by first calculating the average number of genomes for all the 20 mers present in every 500-nucleotide windows across the entire genome and then color each window based on the genome frequency (minimum 1 in yellow and maximum 20 in black). **(B)** was similar to **(A)** but the non-coding regions were masked with light blue color to highlight the oligonucleotide frequencies for the areas that correspond to both forward (upper) and reverse-complement (lower) protein coding sequences. The order of the unfinished genomic contigs was arranged in the same order as appeared in the sequences downloaded from NCBI. The genomes in the plot were ordered based on the 16S rRNA phylogenetic tree (Figure 1) with a dendrogram derived from the same tree to show the relatedness.

The NUCmer plots above are limited to viewing comparisons only between two given genomes. To view and compare nucleotide difference/similarity for all genomes on the same plot, the overall oligonucleotide composition and frequency can be measured along the entire genome and the results can be plotted out and visually compared to each other. This analysis started by collecting all the possible 20-mer sequences in all 20 genomes and then count for each 20-mer how many genomes have each particular sequence. The number of genomes (genomic frequency) for each 20-mer thus ranges from 1 (unique) to 20 (universal). The frequencies can be calculated and plotted along the entire genome by taking every 20-mer from the beginning to the end of the genome. Figure 6A depicts the results of the 20-mer oligonucleotide frequencies among all 20 genomes (including the out-group *P. asaccharolytica* DSM 20707). If a region of a genome is shared by all other 20 genomes, it is colored black; and if a region is unique to the genome itself, it is colored bright yellow. In other words, a black region means that all the possible 20-mer sequences appeared in all tested genomes, whereas the brightest yellow regions have unique 20-mer sequences that are only found in one genome. For easy comparison, the order or the genomes shown in Figures 6A,B were arranged according to that of the *16S rRNA* tree (Figure 1) with a dendrogram reflecting similar tree topology. As expected, the two closest strains ATCC 33277 and 381 share almost identical 20-mer frequency patterns, with the exception of a small insertion at nucleotide position ca. 1,400,000, which is also detected by the NUCmer plot in Figure 5A. The genome of strain 381 is ca. 24 Kbps longer than that of ATCC 33277 due to this insert and the length difference is illustrated in Figure 6A because the length of the bars were based on actual genome sizes. Another interesting example observation is that even though strain JCVI SC001 is closest to SDJ2 due to their identical *16S rRNA* sequences (Figure 1), their oligonucleotide frequency patterns are quite different, with each showing unique regions (brighter colors) at different places. This can be due to two possibilities: (1) the artificial order of the unfinished sequence contigs (in the plots, contig order was the same as that in the downloaded sequences); and (2) the bona fide differences in sequence. This is also true for the other three genomes A7436, W83, and W50, which share identical *16S rRNA* sequences but exhibit distinct frequency patterns.

When the frequencies were plotted only in the open reading frames (ORFs), most of the ORFs appeared dark in color and are shared by the majority of the *P. gingivalis* genomes (Figure 6B). Those ORFs with colors close to yellow should account for the differences of the number of unique proteins previously identified (Figure 3). These differences in nucleotide sequences are essentially reflected in the genes and then translated to proteins, and ultimately accounting for differences in biological functions.

Finally the out-group *P. asaccharolytica* DSM 20707 shows mostly yellow colors in the plot, which is as expected and means that *P. asaccharolytica* does not share much of the 20-mer oligonucleotide sequences with *P. gingivalis*. Interestingly, by lowering the oligomer size to 14 bases (14-mer), most of the DSM 20707 genome appears black, meaning that these two different species share most of the 14-mer sequences (data not shown). When the plot was generated with 15-mer sequences, the *P. asaccharolytica* DSM 20707 genome started to show patches of yellow area (data not shown), indicating that some unique 15-mer sequences are present between these two species. With 15-mer all *P. gingivalis* genomes are black in the plot (data not shown), meaning 15-mer is too short and have not enough resolution power to differentiate unique regions among *P. gingivalis* genomes. Hence whether the oligomer frequency analysis can detect unique/shared regions in a group of genomes, depends on the size of the oligomer. The choice of 20-mer was able to identify unique regions among the strains of *P. gingivalis*, as shown in Figures 6A,B, yet is too sensitive for a different species.

## Comparative functional genomics

The comparative genomics is less meaningful without association with biological functions. Most functional genomic annotations rely on either DNA or protein sequence homology to other sequences with known biological functions. The most popular genome annotation pipeline is probably the NCBI Prokaryotic Genome Annotation Pipeline (Tatusova et al., 2016), which is the current default annotation pipeline when a microbial genome sequence is deposited to and published in NCBI. Several other microbial genome annotation pipelines have also been published and commonly used, including the RAST system—Rapid Annotation of microbial genomes using Subsystems Technology (Overbeek et al., 2014); the BASys—Bacterial Annotation System, a web server for automated bacterial genome annotation (Van Domselaar et al., 2005). Above the gene level, there have also been tools and databases available for constructing and comparing metabolic pathways of microbial genomes. Examples in this category are IMG—the integrated microbial genomes comparative analysis system (Markowitz et al., 2014) and BlastKOALA—a KEGG tool for functional characterization of genome sequences (Kanehisa et al., 2016). Both systems provide annotation information beyond individual gene and protein level, such as, in the case of IMG, conserved protein domain and groups COGs and families (Pfam), as well as the enzymes and metabolic pathways inferred by BlastKOALA.

In this report we compared the *P. gingivalis* genomes at the functional level based on three systems: The NCBI annotation, the RAST annotation, and the BlastKOALA inferred metabolic pathways. The results of these analyses are too voluminous to be presented in text however the complete results are provided in a central online site for download (ftp://www.homd.org/publication_data/20160425). Here we summarized all the comparisons into a single table (Table 8). Initially, functional comparisons were done based on simple text search—by counting the number of genes with functional annotations containing several categories of keywords listed in the table (in Italic font).

**Table 8:** **Comparative functional genomics of *P. gingivalis* genomes^a^^,^^b^^,^^c^**.

**Annotation Source**	**ATCC33277**	**HG66**	**381**	**W83**	**W50**	**A7436**	**AJW4**	**F0570**	**JCVISC001**	**SJD2**	**F0568**	**F0569**	**Ando**	**F0185**	**W4087**	**MP4-504**	**A7A1-28**	**F0566**	**TDC60**	**PaDSM20707**
**GINGIPAIN:** ***rgp, kgp, gingipain***																				
NCBI	6	0	0	0	0	0	0	0	2	0	0	0	2	0	1	0	0	0	5	0
RAST	0	0	0	1	1	1	0	0	0	0	0	0	0	0	0	0	0	0	0	0
BLAST^d^	**7**	**6**	**7**	**5**	**3**	**5**	**6**	**3**	**4**	**2**	**3**	**3**	**5**	**3**	**4**	**4**	**4**	**4**	**6**	**0**
**ATTACHMENT:** ***adhesin, fim, pili, pilus, fimbriae, fimbrilin***																				
NCBI	7	6	5	1	8	4	4	1	12	3	1	1	6	1	1	10	3	1	5	0
RAST	0	0	0	0	0	0	0	0	0	0	0	0	0	0	0	0	0	0	0	0
BLAST	**16**	**17**	**18**	**14**	**11**	**14**	**17**	**12**	**16**	**11**	**13**	**13**	**13**	**13**	**12**	**15**	**15**	**11**	**14**	**0**
**HEME:** ***heme, haga, hagb, hagc, hemaglu, hemoglo***																				
NCBI	4	1	2	4	4	1	2	3	3	1	4	4	4	4	5	1	2	4	4	1
RAST	1	1	1	1	1	1	1	1	1	3	1	2	1	1	1	3	1	1	1	4
BLAST	**10**	**11**	**10**	**8**	**8**	**10**	**10**	**6**	**6**	**7**	**5**	**6**	**7**	**5**	**8**	**8**	**8**	**7**	**10**	**5**
**GENE MOBILITY:** ***transposon, ISPg, transposase, conjugation, insertion element***																				
NCBI	118	68	94	73	20	98	73	25	30	13	35	23	14	26	14	26	25	45	65	32
RAST	46	50	56	48	35	64	69	42	57	48	57	38	28	40	27	87	46	61	51	24
BLAST	**131**	**133**	**139**	**138**	**46**	**149**	**132**	**46**	**71**	**54**	**66**	**45**	**40**	**43**	**34**	**110**	**68**	**72**	**89**	**37**
**TRANSPOSASE, IS5 FAMILY; K07481^e^**																				
KEGG Orthology	47	45	45	13	3	27	16	0	1	1	1	0	3	2	2	2	14	1	22	0
**CAPSULE:** ***capsul***																				
NCBI	2	3	3	3	1	4	4	1	1	3	1	1	3	1	1	4	3	1	2	1
RAST	3	3	3	3	3	3	2	2	3	3	2	2	2	3	3	2	3	3	3	0
BLAST	**6**	**6**	**6**	**6**	**6**	**6**	**5**	**5**	**6**	**6**	**5**	**5**	**5**	**6**	**6**	**6**	**6**	**6**	**6**	**1**
**CRISPR:** ***crispr***																				
NCBI	4	12	11	11	15	15	1	5	0	0	5	12	2	5	5	6	14	10	11	7
RAST	12	12	12	12	12	12	0	6	0	3	5	12	3	5	5	5	11	8	12	7
BLAST	**14**	**14**	**14**	**15**	**15**	**15**	**1**	**6**	**0**	**3**	**6**	**13**	**3**	**6**	**5**	**6**	**14**	**11**	**15**	**7**
CRISPR arrays^f^	3	3	3	4	5	5	2	3	3	3	7	22	3	15	4	3	4	7	5	2
**PHAGE:** ***phage***																				
NCBI	1	1	3	1	6	1	2	10	13	1	9	5	8	8	12	3	1	6	3	1
RAST	3	2	3	4	4	4	4	2	6	5	2	2	6	4	7	3	2	1	1	2
BLAST	**13**	**13**	**15**	**20**	**18**	**19**	**22**	**19**	**25**	**19**	**18**	**13**	**17**	**18**	**25**	**17**	**13**	**13**	**12**	**3**

a Results were compiled based on the NCBI or RAST genome annotations. Total number of proteins containing any of the keywords shown in each category were recorded for each genome and for NCBI and RAST annotations separately. The detail results are provided in the Supplemental Files available from the FTP site: ftp://bioinformatics.forsyth.org/publication_data/20160425/

b *The keyword search was performed in a case-insensitive manner and allowed matching of the partial word*.

c *The order of genomes was based on that similar to the 16S rRNA phylogenetic tree*.

d *BLAST: all the proteins identified by NCBI and RAST were collected and the sequences searched against all the proteins of all 20 genomes using BPLSTP. The numbers (in bold) indicated for each genome are the number of proteins with ≥ 95% sequence identity and ≥ 95% coverage of the query sequences. The numbers were calculated separatly for NCBI and RAST annotated proteins, and the larger number of the two are shown in this table*.

e *The number of proteins related to the IS5 transposase family was identified by the BlastKOALA program (Kanehisa et al., 2016) with the matching to the KEGG Orthology (KO) number K0748*.

f *The number of CRISPR arrays detected by the online software CRISPRfinger (http://crispr.i2bc.paris-saclay.fr/Server/); only the number of “confirmed” candidates were reported thus excluding those “questionable” ones, which only have two DR and one spacer sequences*.

Interestingly marked differences were observed in the NCBI and RAST annotations either by total protein count or by keyword searches. For example, as shown in Table 4, the difference of the total number of protein encoding genes annotated by the two systems can be as large as 351 (for strain F0566). The difference in functional annotation is also quite noticeable (Table 8). For example, only five of the 19 *P. gingivalis* genomes have proteins annotated as “gingipain” by NCBI, whereas three other different genomes were annotated by RAST to have a single gingipain gene.

To remedy the differences and apparent incompleteness of the two annotation systems, a more effective way to detect most, if not all, proteins of the same function, is to perform sequence similarity searches using protein sequences that had been identified. For example, the 19 proteins that were annotated as “gingipain” (16 by NCBI, three by RAST, Table 8) were grouped together and used as the baits to search against all proteins of all 20 genomes identified by both systems. A total of 84 proteins highly similar to the 19 gingipain proteins were detected this way among all 19 *P. gingivalis* genomes ranging from two to seven gingipains per genome (none was detected in *P. asaccharolytica*). These searches were conservative by setting a high percent sequence identity and coverage, and so the numbers can be under-estimated. This approach was done repeatedly for each of the seven functional categories that were deemed of high interest by authors. All the proteins identified by either NCBI or RAST in each category were collectively searched against all protein sequences in all 20 and the number of proteins with ≥ 95% sequence identity and ≥ 95% alignment coverage to the query sequences were recorded. The results of the BLAST searches were listed in the third row of each category in Table 8. Unsurprisingly, the number of genes identified in all categories is higher than those provided by either annotation system, and often higher than both systems combined. The fact that stringent BLAST search identified more proteins of the same function indicates that the currently microbial genome annotation pipelines are quite incomprehensive and are in need of improvement.

For gingipains, using the 16 NCBI identified proteins and three RAST ones (Table 8), the BLAST search of these sequences matched with many more proteins that are highly similar to gingipains in all 19 *P. gingivalis* genomes. Examining the annotation for those proteins highly matched with annotated gingipains, most of them were simply annotated as “hypothetical” or “functionally unknown” proteins, while some were annotated as “peptidase”.

Another notable observation is the high prevalence of the transposase proteins encoded in this species, as high as 149 copies in strain A7436. The lower number of transposases detected in those unfinished genomes is most likely due to the in-between-contig sequence gaps that may contain highly repeated sequences such as the transposases and the IS elements. The completed genome with lowest number of mobility related genes is strain A7A1-28 where only 68 were detected in the genome.

Capsular polysaccharide (CPS) has long been recognized as an important virulence factor for *P. gingivalis* (Singh et al., 2011) and encapsulated strains are known to be more virulent than the non-encapsulated ones (Laine and van Winkelhoff, 1998). When all the annotated capsule related proteins were BLASTP searched against all genomes, the total number of capsule related proteins ranged consistently between five and six copies (Table 8). For example, W83 is known as an encapsulated strain and ATCC 33277 is non-encapsulated. However, both strains encode six copies of capsulated related genes. Of these, four were annotated as “CPS/capsule biosynthesis proteins” by both NCBI and RAST. Interestingly, NCBI only identified three of these four CPS biosynthesis protein. The 5th one was annotated as “CPS transport protein” in W83 by NCBI (Genbank ID AAQ65636.1) but was annotated as “conserved hypothetic protein” in ATCC 33277 by NCBI (BAG34043.1) or “tyrosine-protein kinase Wzc” in both W83 and ATCC 33277 by RAST. The 6th capsule related gene was annotated as “sugar isomerase” in ATCC 33277 (BAG34552.1) or “SIS domain protein” in W83 (AAQ65335.1) by NCBI. This same gene was annotated as “arabinose 5-phosphate isomerase” in both W83 and ATCC 3327 by RAST and “sugar phosphate isomerase involved in capsule formation” in several other strains by NCBI. Taken together, this serves as an example of how inconsistent both annotations are, for genes involved in a single biological function. By BLAST searching using proteins annotated as capsule related genes annotated across all 19 *P. gingivalis* genomes, we were able to detect consistently between five to six copies of genes involved in encapsulation for this species. The fact the all *P. gingivalis* genomes contain a similar number of capsule related genes yet some are encapsulated and others are not, indicates that these genes may subject to different gene expression controls. It is thus likely that some non-encapsulated strains may become encapsulated under certain specific *in vivo* conditions.

In a very different functional aspect, there is a high prevalence of the bacterial phage related proteins, such as phage integrase/site-specific recombinase, phage tail component proteins, and phage-related lysozyme. The number of phage related proteins detected in the 19 *P. gingivalis* genomes ranged from 12 to 25. Functional bacteriophage have so far never been detected in this species (Sandmeier et al., 1993) yet contrarily many proteins related to phage reproduction were detected in all the 19 *P. gingivalis* strains. One most plausible explanation is the prevalence of the CRISPR/Cas systems in this species (discussed below); another is also the presence of the abortive phage infection proteins found in several strains (ATCC 33277, HG66, W83, AJW4, SJD2, and MP4-504, data not shown).

As mentioned above, another very interesting category of enzymes reported in Table 8 is the prevalence of proteins associated with the CRISPR (clustered regularly interspaced short palindromic repeats) elements. CRISPR, together with the Cas (CRISPR associated) proteins, have been dubbed as the adaptive immune system for Bacteria and Archaea to ward off invading foreign DNA (Horvath and Barrangou, 2010). However, although CRISPR arrays were detected in all genomes (including outgroup *P. asaccharolytica*, 4th row in the CRISPR category of Table 8), that is not the case for the Cas proteins. Cas was not detected in the genome of strain JCVI SC001, and only one copy detected in strain AJW4 (3rd row in Table 8 CRISPR category). Strain F0569 has the highest number or CRISPR arrays detected using the online software CRISPRfinger (http://crispr.i2bc.paris-saclay.fr/Server) but this strain does not have the highest number of Cas proteins. Of all the CRISPR arrays detected, the length of the direct repeat (DR) element ranged from 23 to 47 bps and the number of the DRs in the array ranged from 5 to 121 copies (data not shown but available from the online FTP site). Both ATCC 33277 and strain 381 had three copies of nearly identical CRISPR arrays and both had one copy of the arrays with 121 DR sequences (and 120 spacer sequences). The high DR copy number may be an indication for the CRISPR activity in the past. On the other hand, JSVI SC001 had three copies of CRISPR arrays detected with DR of 31, 26, and 45 bps and repeat number 5, 7, and 6 respectively. Whether or not this strain possesses a type of Cas protein that is very different from those in other strains remains to be investigated. If this strain lacks any functional Cas protein, it is likely to be susceptible to bacteriophage infection or the activation of the possible presence of prophages as evidenced by the detection of 25 copies of phage related proteins (Table 8).

On the other side of the scale, at the metabolic pathway level, the KEGG pathways and KEGG Orthology identified by BlastKOALA are BLAST-based, i.e., all the proteins sequences regardless of their annotations, were BLAST-searched against the online protein database used by BlastKOALA (http://www.kegg.jp/blastkoala/). Hence the comprehensiveness of the KEGG pathways and the KO terms inferred by BlastKOALA depend on the completeness of the proteins in the database. At any rate, several metabolic pathways identified to be unique to the species *P. gingivalis* are: Glycosphingolipid biosynthesis–globoseries; sphingolipid metabolism; lysosome; glycosphingolipid biosynthesis—ganglio series; and glycosaminoglycan degradation. These species specific pathways were suggested based on the fact that they were detected in all of the 19 *P. gingivalis* genomes but not in *P. asaccharolytica* DSM 20707 (detailed data available in the FTP site). When compared to *P. asaccharolytica* DSM 20707, BlastKOALA determined that *P. gingivalis* lacks proteins involved in the following pathways: C5-branched dibasic acid metabolism; AMPK signaling pathway; amoebiasis; thyroid hormone synthesis; apoptosis; and arachidonic acid metabolism. Note that the specific-specific observations made above were only based on using a different species of the same genus. Inclusion of more outgroups would certainly reduce the number of species-specific functions or pathways.

## Comparison with other species

The focus of this paper is the within-species comparison for the different strains of *P. gingivalis* (PG). It is however also very interesting and important to compare to other related species. Due to the great amount of data generated already from the within-species comparisons, a full multi-species comparison study will be reported in a separate publication. Here we present only a brief summary of the comparison of *P. gingivalis* genomes with several selected species (Table 9).

**Table 9:** **Genome statistics of selected species^a^**.

**Category**	**PG**	**TF**	**TD**	**AA**	**BU**	**BF**
Number of genomes	19	7	17	38	16	103
Number of genomes with single contig	9	3	6	8	0	4
Number of contigs (genomes with > 1 contig)	92–192	71–141	2–12	4–787	8–343	2–2,566
Genome sizes	2,216–2,441 kb	3,233–3,405 kb	2,742–2,990 kb	1,860–2,382 kb	4,270–5,047 kb	4,457–8,029 kb
Mean genome size	2,320 kb	3,312 kb	2,838 kb	2,155 kb	4,771 kb	5,416 kb
Number of ORFs^b^	1,788–2,417	2,492–3,001	2,520–2,793	1,829–2,364	3,254–4,663	3,593–8,060
Mean ORF number	2,101	2,740	2,634	2,046	4,045	4,760
Number of core proteins^c^	1,037	1,560	1,129	424	1,191	NA
Number of unique proteins^d^	1,044	801	692	1,233	4,040	NA
Non-hypothetical proteins^e^ (percentage)	1,206–1,637 (54.16–74.26%)	1,573–1,800 (58.77–64.65%)	685–1,618 (25.17–58.47%)	1,127–2,035 (48.43–89.75%)	1,038–3,156 (24.84–79.48%)	677–5,732 (13.99–71.91%)
Non-hypothetical proteins: Mean (Percentage)	1,346 (64.60%)	1,702 (62.18%)	801 (30.38%)	1,656 (81.19%)	2,450 (60.71%)	2,993 (62.63%)
Hypothetical proteins^e^ (Percentage)	515–1,108 (25.74–45.84%)	919–1,201 (35.35–41.23%)	1,149–2,090 (41.53–74.83%)	211–1,200 (10.25–51.57%)	784–3,149 (20.52–75.16%)	1,193–4,162 (28.09–86.01%)
Hypothetical proteins: Mean (Percentage)	755 (35.40%)	1,038 (37.82%)	1,832 (69.62%)	390 (18.81%)	1,594 (39.29%)	1,767 (37.37%)
**UNIQUE RASE SUBSYSTEM^f^**						
Level 1		1				NA
Level 2		1	1	6	1	NA
Level 3	4	4	15	37	5	NA
Level 4	25	28	141	397	84	NA
**MISSING RAST SUBSYSTEMS^f^**						
Level 3		1				NA
Level 4		11				NA

a *PG, Porphyromonas gingivalis; TF, Tannerella forsythia; TD, Treponema denticola; AA, Aggregatibacter actinomycetemcomitans; BU, Bacteroides uniformis; BF, B. fragilis Genomes are downloaded from the NCBI FTP site; only those genomes with annotation were analyzed*.

b *Number of NCBI predicted ORFs based on the number of proteins found in the “.faa” file for each genome*.

c *Number of core proteins are those present in all genomes of the species, identified with the “blastclust” software (https://www.ncbi.nlm.nih.gov/Web/Newsltr/Spring04/blastlab.html) using 95% sequence identity and 90% length coverage as the parameters*.

d *Number of unique proteins are those present in a single genome of the species, identified with the “blastclust” software using 60% sequence identity and 50% length coverage as the parameters*.

e *Non-hypothetic proteins predicted by NCBI, are proteins with annotation that does not contain the key words “hypothetic” and “uncharacterized”; hypothetical ones are those with annotation that contains either words*.

f *The 4 levels of subsystems were defined by the RAST (Aziz et al., 2008); the unique systems for a species (regardless of levels) are those that can be found in ≥ 85% of all the genomes in the species, and < 15% in all other species; the missing subsystems are those found in < 15% of the target species but present in ≥85% of all other species. The blank cells are those with none found; NA: not analyzed. The 85/15% cutoff was chosen based on the number of genomes in TF so that it allowed detection of a subsystem present or missing in only 1 out of 7 genomes*.

*P. gingivalis* originally belonged to the genus *Bacteroides* but was reclassified to the new genus *Porphyromonas* due to its marked biochemical and chemical differences (Shah and Collins, 1988). Based on the 16S rRNA phylogeny the closest bacterial genus found in human oral cavity is *Tannerella*, which was also re-classified from *Bacteroides* (Sakamoto et al., 2002). *Bacteroides* is the next closest genus to *Porphyromonas*. Naturally it is highly interesting and important to compare genomes of these close genera. So far genomics of seven strains of the species *T. forsythia* (formerly *T. forsythensis*) (TF) have been sequenced. As to *Bacteroides*, the most sequenced species is *B. fragilis* (BF), an important human gut bacterium, with a total of 114 strains/genomes sequenced to-date (only 103 were annotated). The runner-up is *B. uniformis* (BU), another human gut bacterial species with 21 genomic sequences available (16 were annotated). However, both of these two gut *Bacteroides* species are not considered human oral species. The only oral *Bacteroides* species that has been sequenced is *B. pyogenes* (BP), with five sequenced genomes that actually belong to four different strains (strain DSM 20611 = JCM 6294 was sequenced twice separately).

Table 9 also includes summary statistics for two other important human oral pathogenic species—*Treponema denticola* (TD) and *Aggregatibacter actinomycetemcomitans* (AA). It has long been recognized that bacterial species exist in complexes in subgingival plaque. One complex was found by Socransky et al. (1998) to consist of the tightly related group *P. gingivalis, T. denticola*, and *T. forsythia*. This complex related strikingly to clinical measures such as pocket depth and bleeding on probing in chronic periodontitis. *A. actinomycetemcomitans* has for decades been associated with aggressive forms of periodontitis in adolescents (Haubek and Johansson, 2014).

Of the six species summarized in Table 9, *A. actinomycetemcomitans* has the smallest mean genome size (2,155 kb) and *B. fragilis* the largest (5,416 kb). The average number of protein genes encoded by these genomes are as expected proportional to the genome sizes, from 2,046 to 4,760 ORFs for AA and BF respectively. Using the most stringent criteria to define the core and unique proteins used for *P. gingivalis* in this study, *T. denticola* and *B. uniformis* both have similar numbers of core proteins when compared to PG. *T. forsythia* appeared to have higher number of core proteins (1,560). This is most likely due to the lower number of genomes analyzed) and that if more genomes are sequenced fewer core proteins would be identified. AA appeared to have the lowest number of core proteins detected but it also has twice the number of genomes analyzed compared to PG, TD, and BU (38 vs. 19, 17, and 16). We were not able to analyze the 103 BF genomes using the “blastclust” software due to the extreme slow speed with larger number of genomes. It would otherwise be interesting to examine the number of core proteins detected using the same criteria (95% sequence identity and 90% length coverage) for this large group of genomes. Similarly the number of unique proteins detected (with 60% sequence identity and 60% length coverage) is also, if not completely, determined by the size and number of genomes used for the analysis. The larger the sizes of the genomes, or the fewer the number of the genomes used for analysis, the more unique proteins were detected.

For functional comparison, Table 9 summarizes the total number of non-hypothetical and hypothetical proteins annotated by NCBI based on the absence or presence of either keyword “hypothetical” or “uncharacterized” associated with the protein annotation. The average percentage of non-hypothetical proteins differs greatly between species from 30.38% in TD to as high as 81.19% in AA (69.62% and 18.81% respectively for hypothetical proteins). A preliminary functional comparison based on RAST annotation for these genomes revealed several unique higher level subsystems. For example, the unique “Level 2” RAST subsystem identified in TD is “Motility and Chemotaxis” which is also expected because TD is a highly motile spirochaete species. The only Level 1 subsystem determined by this analysis is “Secondary Metabolism—Lanthionine biosynthesis” which is associated with the LanB and LanC proteins identified in TF. Lanthionine has been found in bacterial cell walls and is also a component of a group of genes encoding peptide antibiotics called lantibiotics. They are a type of bacteriocins commonly found and made by different genera of actinomycetes (Maffioli et al., 2015). The true roles of the lanB and lanC genes identified in TF may be worth investigating. For PG, there are four Level 3 subsystems detected that are unique and not present in the other 5 species: (1) Amino Acids and Derivatives—Lysine, threonine, methionine, and cysteine—Lysine fermentation; (2) Amino Acids and Derivatives—Proline, 4-hydroxyproline uptake and utilization; (3) Stress Response—Dimethylarginine metabolism; and (4) Virulence, Disease and Defense—Resistance to antibiotics Vancomycin. The vancomycin resistance is conferred by a gene encoding vancomycin B-type resistance protein VanW, found exactly 1 copy in all of the 19 PG genomes, but not in all other genomes of other species.

Above is only a very brief description of what were identified as unique or missing functions among this selected group of species based on a very preliminary analysis. More data from which Table 9 was derived can be found in the FTP site dedicated to this publication ftp://ftp.homd.org/publication_data/20160425/8_Comparison_to_other_species/. A more comprehensive comparative genomics study for these, and more interesting species and genomes is under investigation and will be reported in a separate publication in the future.

## Concluding remarks

In this report 19 genomes of the species *P. gingivalis* as well as the outgroup species *P. asaccharolytica* were compared at several different levels of information ranging from nucleotide to genes to proteins and metabolic functions. Based on the single gene 16S rRNA phylogeny and multi-gene pholygenomic approach using core/shared protein sequences, several plausible evolutionary paths were suggested. Although there is no single evolutionary path concluded by these analyses, two closely related groups were consistently observed throughout the analyses. The first group consists of strains ATCC 33277, 381, and HG66 and the second of W83, W50, and A7436. The group of ATCC 33277, 381, and HG66 is also closer to the possible common ancestor inferred based on the use of an outgroup species *P. asaccharolytica*. We also detected at least 1,037 core/shared proteins for this species based on 95% sequence similarity and 90% alignment length. However, the number of core proteins increases with the lowering of the two detecting parameters. Functional and metabolic pathways were also compared and suggested several important functions of pathways that are unique to this species, to each strain, or missing in any particular strain. *P. gingivalis* has many genes encoding proteins related to or involved in gingipains, attachment (e.g., adhesins and fimbrins), capsules, and phages. These proteins were either missing or present in very few copies in the neighbor species *P. asaccharolytica*. Particularly intriguing observations were prevalence of many proteins related in phage productions and the equal prevalence of the CRISPR system in this species, with the exception of one strain lacking the Cas proteins.

Despite the large amount of comparative results generated in this study, there are still many different ways and software tools for analyzing and comparing a group of genomes. The complete results presented in this report, together with several other results that were only mentioned briefly here, are made available for download online at ftp://www.homd.org/publication_data/20160425/. We hope these data are useful to the research community and more hypotheses can be formulated based on the current or future analyses in order to gain deeper understanding on this important periodontal pathogen.

## Author contributions

TC: Data acquisition, data analysis, data interpretation, writing of the manuscript, final approval of the version to be published; HS: Data acquisition, data analysis, data interpretation, writing; IO: Initiating the study, writing of the manuscript, revising the manuscript, final approval of the version to be published.

## Funding

This work was supported by The Forsyth Institute Bioinformatics Core and the European Commission (FP7-HEALTH-306029 “TRIGGER”).

### Conflict of interest statement

The authors declare that the research was conducted in the absence of any commercial or financial relationships that could be construed as a potential conflict of interest. The reviewer DY and handling Editor declared their shared affiliation and the handling Editor states that the process nevertheless met the standards of a fair and objective review.
